# Standard Sample Storage Conditions Have an Impact on Inferred Microbiome Composition and Antimicrobial Resistance Patterns

**DOI:** 10.1128/Spectrum.01387-21

**Published:** 2021-10-06

**Authors:** Casper Sahl Poulsen, Rolf Sommer Kaas, Frank M. Aarestrup, Sünje Johanna Pamp

**Affiliations:** a Research Group for Genomic Epidemiology, National Food Institute, Technical University of Denmark, Kongens Lyngby, Denmark; Lerner Research Institute

**Keywords:** microbiome, sample storage, freezing, metagenomics, microbial community composition, complex samples, spiking, synthetic mock community, antimicrobial resistance, bacteria, mock community, parasite

## Abstract

Storage of biological specimens is crucial in the life and medical sciences. Storage conditions for samples can be different for a number of reasons, and it is unclear what effect this can have on the inferred microbiome composition in metagenomics analyses. Here, we assess the effect of common storage temperatures (deep freezer, −80°C; freezer, −20°C; refrigerator, 5°C; room temperature, 22°C) and storage times (immediate sample processing, 0 h; next day, 16 h; over weekend, 64 h; longer term, 4, 8, and 12 months) as well as repeated sample freezing and thawing (2 to 4 freeze-thaw cycles). We examined two different pig feces and sewage samples, unspiked and spiked with a mock community, in triplicate, respectively, amounting to a total of 438 samples (777 Gbp; 5.1 billion reads). Storage conditions had a significant and systematic effect on the taxonomic and functional composition of microbiomes. Distinct microbial taxa and antimicrobial resistance classes were, in some situations, similarly affected across samples, while others were not, suggesting an impact of individual inherent sample characteristics. With an increasing number of freeze-thaw cycles, an increasing abundance of *Firmicutes*, *Actinobacteria*, and eukaryotic microorganisms was observed. We provide recommendations for sample storage and strongly suggest including more detailed information in the metadata together with the DNA sequencing data in public repositories to better facilitate meta-analyses and reproducibility of findings.

**IMPORTANCE** Previous research has reported effects of DNA isolation, library preparation, and sequencing technology on metagenomics-based microbiome composition; however, the effect of biospecimen storage conditions has not been thoroughly assessed. We examined the effect of common sample storage conditions on metagenomics-based microbiome composition and found significant and, in part, systematic effects. Repeated freeze-thaw cycles could be used to improve the detection of microorganisms with more rigid cell walls, including parasites. We provide a data set that could also be used for benchmarking algorithms to identify and correct for unwanted batch effects. Overall, the findings suggest that all samples of a microbiome study should be stored in the same way. Furthermore, there is a need to mandate more detailed information about sample storage and processing be published together with DNA sequencing data at the International Nucleotide Sequence Database Collaboration (ENA/EBI, NCBI, DDBJ) or other repositories.

## INTRODUCTION

Metagenomics has emerged as an important technology to reveal microbiome composition at the taxonomic and functional levels and in the context of where they reside, such as in human and animal hosts and in the environment. The sample processing workflow in metagenomics comprises a number of steps, including sample collection, sample storage, DNA isolation, library preparation, DNA sequencing, and data analysis. In microbiome analyses, there is an increasing concern about the influence of sample processing on skewing inferred microbial community composition, which may explain differences observed between studies (1–5). Increasingly, data from different metagenomic projects are combined as part of a study without taking confounding factors such as differences in sample processing into account. Therefore, it is currently uncertain to what extent it is in fact possible to compare data across studies in a meaningful way without leading to misleading conclusions. Comparisons of data across laboratories and projects are of great importance and could facilitate meta-analyses of human, animal, and environmental microbiome studies as well as a global metagenomics-based surveillance of pathogens and antimicrobial resistance (6, 7).

Standardization of sample storage conditions can be difficult because sample collection may be performed over a long period of time, suitable storage facilities may be unavailable, for instance, in field studies, and proper shipping conditions can be limited when samples need to be transported to a central laboratory for analysis (8, 9). A number of studies involving 16S rRNA gene profiling have investigated aspects of storage conditions on the fecal microbiota (10–13). From these studies, different variables affecting the microbial composition were identified, such as the temperature and duration of sample storage and the addition of preservation liquids (10–13). Some studies have concluded that storage has a limited effect on inferred microbial community composition relative to the variation observed between different samples (10, 11). However, this may depend on how different microbiomes initially were between samples according to certain measures and the kind of secondary analyses and interpretations that were applied.

The role of storage conditions in metagenomics-based studies is less well described. In a study involving throat swabs, two time points were investigated, namely, 0 h (i.e., DNA isolation immediately following sample arrival at the lab) and after 24 h of storage at room temperature (5). The study revealed an altering of the microbial community composition, and an increase in the bacterial ratio to human DNA was observed that was hypothesized to be the result of bacterial growth. More detailed studies are needed to determine the influence of relevant sample storage conditions on inferred microbial community composition of microbiomes.

Here, we assess the effect of storage temperature and time on two different sample types (pig feces and domestic sewage), represented by two samples per same type, respectively. We further investigate the effect of repeated freeze-thaw cycles on inferred microbiome composition. We find that storage has a systematic effect on microbiome composition, resulting from abundance changes of distinct microbial genera. However, the effect on specific genera was difficult to generalize across different sample types. A high correlation was observed between patterns of taxonomic microbial community structure and the antimicrobial resistome pattern. Based on our findings, we provide recommendations for sample storage conditions.

## RESULTS

**FIG 1:**
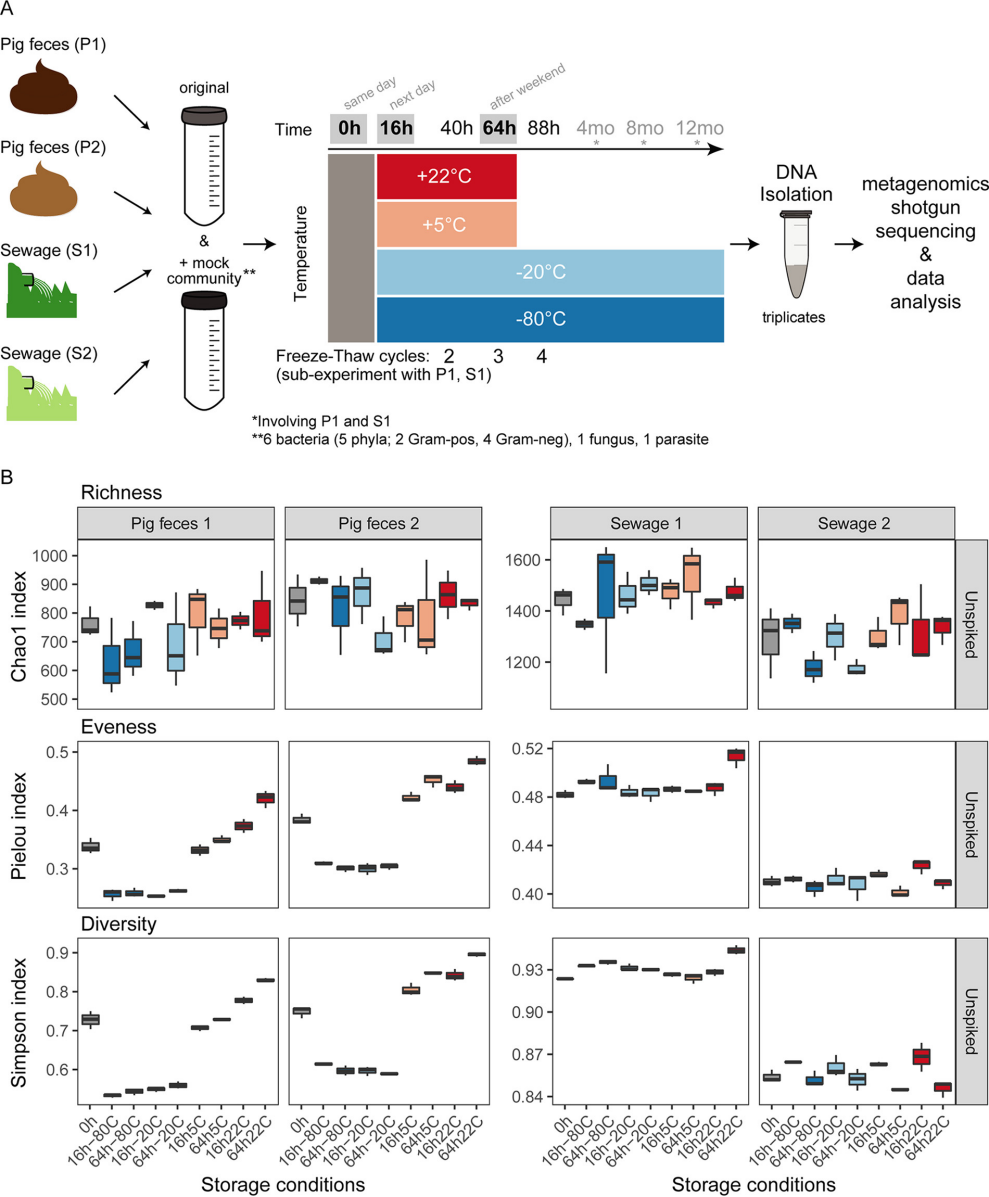
Overview of the experimental setup and alpha diversity of microbiomes. (A) Two pig feces and two sewage samples were divided into two large aliquots, of which one was spiked with a synthetic mock community composed of eight microorganisms that included two eukaryotes (Propionibacterium freudenreichii, Bacteroides fragilis, Staphylococcus aureus, Fusobacterium nucleatum, Escherichia coli, Salmonella enterica serovar Typhimurium, Cryptosporidium parvum, and Saccharomyces cerevisiae), respectively. Each of these eight large aliquots was divided into small aliquots that were stored at four different temperatures (−80°C, −20°C, 5°C, and 22°C) for six different durations of time (0 h, 16 h, 64 h, 4 months, 8 months, and 12 months). DNA was isolated at the three different time points in triplicate. Additional aliquots for samples P1 and S1 underwent repeated freeze-thaw cycles following 40 h, 64 h, and 88 h of storage duration. For details, see Materials and Methods. All samples were sequenced on an Illumina HiSeq. (B) Alpha diversity was determined (richness [Chao1], evenness [Pielou’s evenness], and diversity [Simpson]). The indices were calculated from the count table aggregated at the genus level. For the results of the duplicate sets of samples that also harbored the spiked mock community, see Fig. S3 in the supplemental material.

In order to determine the effect of relevant microbiome sample storage conditions on different sample types, two pig feces (P1 and P2) and two sewage (S1 and S2) samples were investigated. These samples were processed and analyzed individually as pure sample material as well as material spiked with a synthetic microbial community composed of eight different microorganisms (Fig. 1A; see also Tables S1, S2 and S3 in the supplemental material). Aliquots of all four samples (P1, P2, S1, and S2) were exposed of combinations of 9 different storage conditions: four temperatures (−80°C, −20°C, 5°C, and 22°C) and three storage duration times (0 h, 16 h, and 64 h) and, in addition, two frozen temperatures (−80°C and −20°C) for three additional storage duration times (4 months, 8 months, and 12 months) for samples P1 and S1. Triplicate DNA isolations were performed for each storage condition. This experiment amounted to a total of 304 pig feces and sewage samples. Furthermore, additional aliquots from samples P1 and S1 that were stored at −80°C and −20°C underwent up to four freeze-thaw cycles (Fig. 1A; Table S1) In addition, 39 control samples were analyzed that included DNA isolation blank controls and the pure mock community. On average, 14.8 million reads were obtained per microbiome sample, with a larger output obtained from the pig feces samples (average of 19.9 ± 8.4 million reads) than from the sewage samples (average of 8.7 ± 3.3 million reads).

Initially, we analyzed all samples from all time points (0 h, 16 h, 64 h, 4 months, 8 months, and 12 months) together (e.g., Fig. S1). This analysis suggested that inferred microbiome composition differed between storage conditions. However, the reasons for some of the patterns were inconclusive because they may also be related to technical variation; the samples from the first three time points (0 h, 16 h, and 64 h) were processed using a different library preparation kit than what was used for the samples from the later time points (4 months, 8 months, and 12 months) (see Materials and Methods). This prompted us to perform a separate study on assessing the effects of library preparation on inferred microbial community composition involving DNA extracts from the same pig feces and sewage samples from the present study (see reference 14). Here, we attempted different strategies of correcting for the underlying batch effects and did not find a way that led to results we could rely on with confidence. Therefore, we focused our analysis in this study on the first time points (0 h, 16 h, and 64 h) as well as the subexperiment involving a series of freeze-thaw cycles, all undergoing the same library preparation and sequencing strategy (i.e., in all 287 samples). In addition, we made accessible under accession number PRJEB31650 the complete metagenomic data sets of the 343 samples presented in this study as well as 95 samples processed with different combinations of library preparation methods and sequencing platforms with detailed metadata (Table S1). This data set of 438 samples (total of 777 Gbp, 5.1 × 10^9^ reads) could, for example, be used to benchmark different algorithms adjusting for batch effects.

### Impact of storage conditions on microbial community richness, evenness, and diversity.

To assess the level of sequencing depth in relation to the genera observed, rarefaction analyses were performed. The rarefaction curves of individual samples (P1, P2, S1, and S2) had similar profiles in which a plateau was approached for most samples, suggesting that the sequencing depth was adequate (Fig. S2).

Overall, the pig feces (P1 and P2) and sewage samples (S1 and S2) exhibited similar richness (Chao1), evenness (Pielou), and diversity (Simpson) whether they were spiked or not spiked with the mock community (Fig. 1B; Fig. S3). Richness (Chao1) was similar, independent of storage condition, for the two pig feces samples and two sewage samples. In contrast, for the pig feces samples, evenness (Pielou) and diversity (Simpson) were lower under frozen storage conditions (−20°C and −80°C), while evenness was higher when samples were stored at temperatures above freezing (5°C and 22°C) than when samples were not stored and were processed immediately (0 h) (Fig. 1B; Fig. S3). This was not observed for the sewage samples, for which evenness (Pielou) was similar across all conditions and slightly increased when stored at 22°C for 64 h. This was also reflected in the Simpson’s diversity index that takes both richness and evenness into account. In summary, storage conditions had an effect on microbial community evenness (Pielou) and diversity (Simpson) in the pig feces samples and had a lower effect on the sewage samples.

### Samples cluster according to their origin.

**FIG 2:**
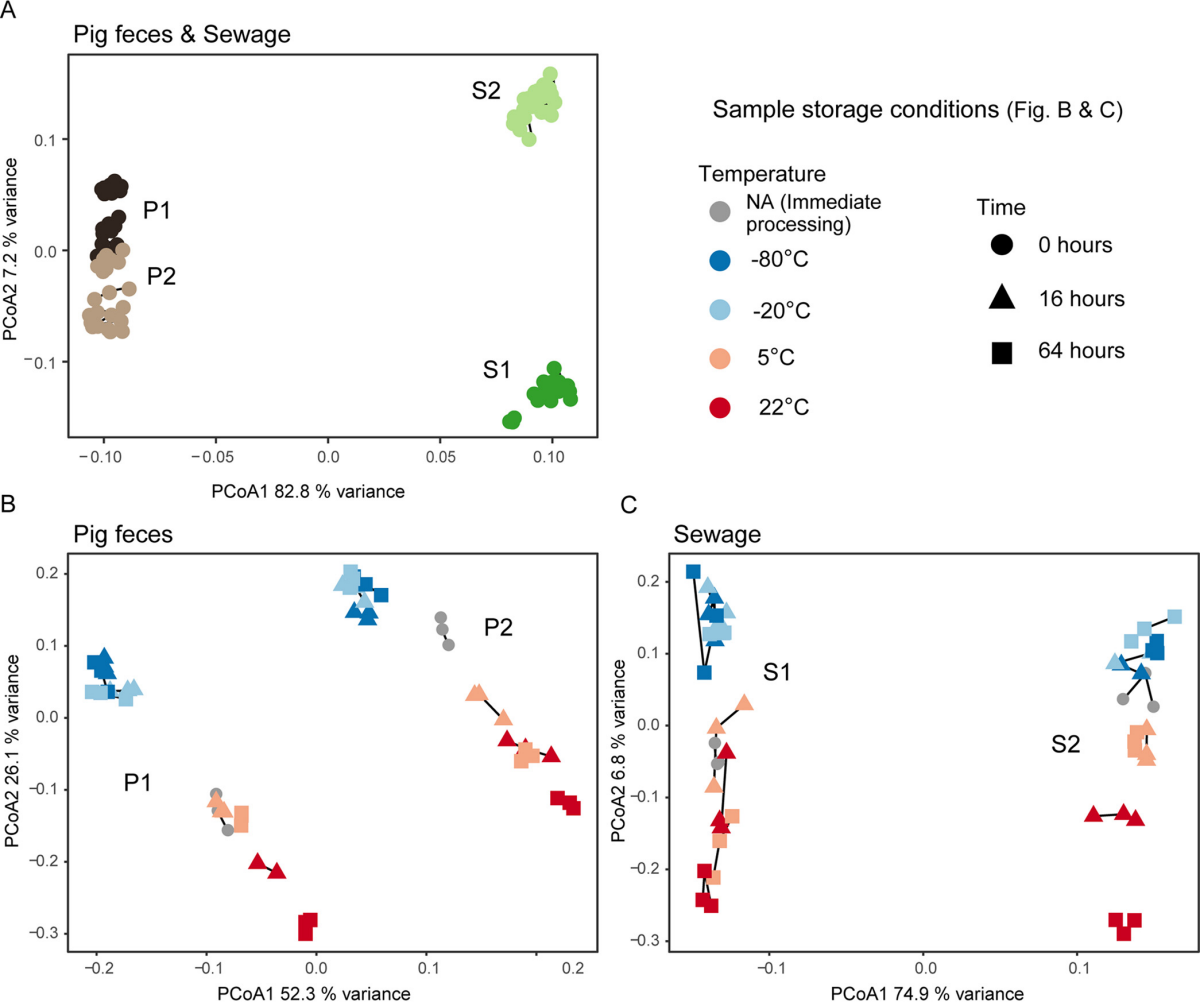
Principal-coordinates analysis (PCoA) of all unspiked samples (A), all unspiked pig fecal samples (B), and all unspiked sewage samples (C). Bray-Curtis dissimilarities were calculated on Hellinger transformed data. The vegan function capscale was used to perform PCoA on the dissimilarity matrix. Variances explained by the two first axes are indicated. Sample replicates are connected with lines. For the results of the individual spiked samples as well as box plot visualizations, see Fig. S4 and S5 in the supplemental material.

A pairwise comparison of all samples was performed by calculating Bray-Curtis dissimilarities on Hellinger transformed data and then visualized using principal-coordinates analyses (PCoA) and box plots (Fig. 2A; Fig. S4). The 212 samples clustered largely according to the sample matrix they originated from (i.e., P1, P2, S1, and S2). The two pig feces samples appeared more similar to each other than the two sewage samples. A separate two-dimensional representation of the pig feces samples (P1 and P2) revealed, however, that they could be discriminated (Fig. 2B; Fig. S5). A clear separation of the two sewage samples (S1 and S2) was also observed (Fig. 2C; Fig. S5). This indicates that the examined storage conditions did not influence the ability to discriminate between the two different pig feces samples and the two different sewage samples, respectively (Fig. 2B and C).

The pairwise dissimilarities for all samples were statistically assessed with “adonis”, and a significant difference between samples was observed (*P* < 0.0001) (Table S4). Since there were significant differences in group homogeneities, the analysis was supplemented by comparing dissimilarities with a Kruskal-Wallis test (Table S5). The Kruskal-Wallis test also revealed a significant difference between samples (*P* < 0.0001) and that samples originating from the same input samples (P1, P2, S1, and S2) were most similar to each other (Table S5).

### A systematic effect on microbiome composition based on sample storage condition.

As evident from the PCoAs, samples clustered in a systematic manner according to storage conditions for both pig feces and sewage (Fig. 2B and C). The frozen samples (−80°C and −20°C) clustered together and separately from the samples processed immediately (0 h) and those stored at higher temperatures (5°C and 22°C). Of note, the microbiomes of the frozen samples remained stable over time (Fig. 2B and C; Fig. S5). In contrast, the microbiomes of samples stored at temperatures over 0°C differed dependent on the exact temperature that they were stored at (i.e., the refrigerator [5°C] and room temperature [22°C]). They also differed from samples processed right after collection (0 h), and it was evident as the microbial communities of these samples (5°C and 22°C) changed along a time gradient in the opposite direction of the frozen samples (−80°C and −20°C) (Fig. 2B and C; Fig. S5). However, the samples stored in the refrigerator (5°C) for 16 h were, in most cases, relatively similar to the samples that were immediately processed (0 h). A relatively large proportion of the variance was represented by the first axis, but more so for the pig feces samples than for the sewage samples. The fact that storage condition had an effect on community composition was also confirmed in the “adonis” test (*P* < 0.0001) after subsetting to sample level (P1, P2, S1, and S2). Model assumptions were in all cases fulfilled (*P* > 0.05) (Table S4).

The average dissimilarities of samples stored under different conditions relative to no storage (0 h; immediate DNA isolation following sample collection) revealed that the samples stored at 22°C for 64 h had the largest dissimilarity relative to immediate DNA isolation except for pig feces sample 1 (Table 1). In general, a large dissimilarity was observed for the frozen samples relative to 0 h, but all frozen samples had a relatively small dissimilarity compared with each other. Samples stored at 5°C for 16 h mostly resembled samples that were processed immediately (Table 1; Table S4). These findings were in agreement with the PCoA.

**TABLE 1:** Average dissimilarities and dispersion (standard error) of stored samples relative to samples that were not stored

	Pig feces 1	Pig feces 2	Sewage 1	Sewage 2
0 h^*a*^	0.066 (0.003)	0.081 (0.003)	0.073 (0.003)	0.057 (0.003)
16 h, −80°C	**0.159 (0.005)** ^*b*^	**0.116 (0.003)**	**0.103 (0.002)**	0.077 (0.002)
64 h, −80°C	**0.152 (0.005)**	**0.113 (0.002)**	**0.121 (0.004)**	0.076 (0.001)
16 h, −20°C	**0.141 (0.004)**	**0.124 (0.003)**	**0.109 (0.002)**	0.082 (0.003)
64 h, −20°C	**0.139 (0.004)**	**0.126 (0.002)**	**0.109 (0.002)**	0.088 (0.002)
16 h, 5°C	0.073 (0.003)	0.098 (0.004)	0.089 (0.008)	0.061 (0.003)
64 h, 5°C	0.098 (0.002)	**0.137 (0.004)**	0.091 (0.003)	0.073 (0.001)
16 h, 22°C	0.096 (0.002)	**0.130 (0.004)**	0.087 (0.002)	0.082 (0.002)
64 h, 22°C	**0.142 (0.002)**	**0.180 (0.003)**	**0.127 (0.002)**	**0.126 (0.001)**

a 0 h, DNA isolation immediately following sample collection; entries for 0 h represent the variation among replicates.

b The numbers in bold represent the samples that are most dissimilar to time point 0 h, respectively (and above 0.1).

### Distinct taxa are driving the differences observed for different storage conditions.

**FIG 3:**
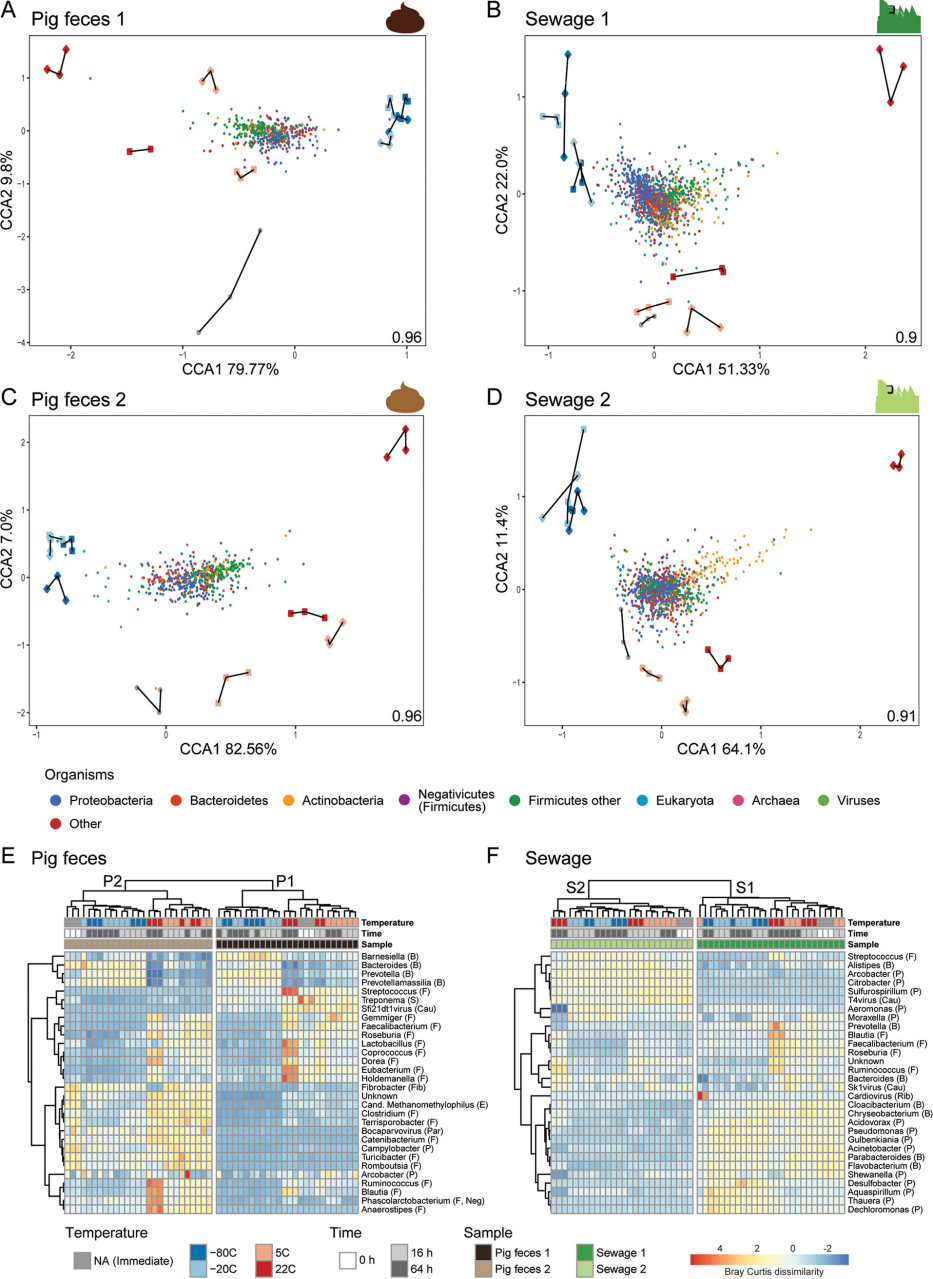
Overview of community composition in relation to storage conditions. (A to D) Ordination plots of canonical correspondence analyses (CCA) of the individual unspiked samples (P1, P2, S1, and S2) visualizing different groups of organisms (colored points) constrained by the storage conditions (colored shapes with replicates connected with lines). The inertia constrained by the explanatory variables is indicated in the lower right corner of the CCA plot, respectively. Heat maps displaying the 30 most abundant genera in unspiked pig feces (E) and unspiked sewage (F) using Bray-Curtis dissimilarity matrix calculated on Hellinger transformed data. Complete-linkage clustering was used for both genera and samples. The clustering of genera was performed on Pearson correlation coefficients and samples. For heat map visualization, genera were standardized into zero mean and unit variance. Phyla are indicated as B (*Bacteroidetes*), F (*Firmicutes*), S (*Spirochetes*), Cau (*Caudovirales*), Fib (*Fibrobacteres*), E (*Euryarchaeota*), Par (*Parvoviridae*), P (*Proteobacteria*), Neg (*Negativicutes*), and Rib (*Riboviria*). For the results of the individual spiked samples, see Fig. S6 in the supplemental material.

In order to determine the microorganisms that changed in abundance due to the different storage conditions, a constrained ordination was performed using canonical correspondence analysis (CCA) (Fig. 3A to D). A general trend across the four sample matrices was observed in that the majority of taxa related to the phyla *Firmicutes* and *Actinobacteria* were associated with samples stored at temperatures above freezing (5°C and 22°C). In contrast, *Bacteroidetes* and *Proteobacteria* were more associated with the frozen samples (−80°C and −20°C) (Fig. 3A to D; Fig. S6).

To elucidate patterns in community structure in more detail, a cluster analysis of pig feces and sewage samples was performed at the genus level and visualized in heat maps (Fig. 3E and F). The two pig feces and two sewage samples clustered separately, supporting the observations from the PCoA. In agreement with the CCA, the cluster analysis of the pig feces samples also revealed that *Firmicutes*, such as *Faecalibacterium*, *Coprococcus*, *Eubacterium*, *Ruminococcus*, and *Blautia*, were more abundant in samples stored at temperatures above freezing (5°C and 22°C) than in samples stored under other storage conditions (Fig. 3A, C, and E). Furthermore, the observation for the pig feces samples that *Bacteroidetes* and *Proteobacteria* were more abundant in frozen samples was also confirmed in part by the cluster analysis in which genera such as *Barnesiella*, *Bacteroides*, *Prevotella*, and *Prevotellamassilia* were observed in higher abundance than in the other samples (Fig. 3A, C, and E). The patterns related to *Firmicutes*, *Bacteroidetes*, and *Proteobacteria* in the CCA compared to the cluster analysis were less clear for the sewage samples (Fig. 3B, D, and F). Some *Firmicutes* genera indeed appeared to be present in higher abundance in samples stored at temperatures above freezing, such as *Blautia*, *Faecalibacterium*, *Roseburia*, and *Ruminococcus*, than what was observed under the other storage conditions (Fig. 3F). Overall, for the sewage samples, it was more difficult to reveal which genera were driving the differences between storage conditions using this type of analysis.

In the CCA, we observed that *Firmicutes* and *Actinobacteria* abundances were associated with storage at temperatures above freezing and that *Bacteroidetes* and *Proteobacteria* abundances were associated with frozen samples (Fig. 3). We assessed this finding further using MA plots with log_2_ fold change values from a differential abundance analysis using DESeq. We focused on storage conditions for which the highest dissimilarities were observed among each other (i.e., samples stored at 22°C, 5°C, and −80°C for 64 h, respectively, compared to immediate DNA isolation [Fig. S7 to S9]). The most striking patterns were observed when comparing samples stored at 22°C for 64 h with samples undergoing immediate DNA isolation (Fig. S7). *Firmicutes* and *Actinobacteria* were observed at higher abundances following storage at 22°C for 64 h, and a corresponding lower abundance was observed for *Bacteroidetes* and *Proteobacteria* than in samples processed immediately (Fig. S7). The *Negativicutes* as part of *Firmicutes* tended to exhibit similar abundance patterns as *Firmicutes*. The effect also seemed independent of abundance with an expected larger variance of the lower abundant genera (Fig. S7). The same patterns were observed when comparing immediate DNA isolation with the samples stored at 5°C for 64 h (Fig. S8). Comparing immediate DNA isolation with samples stored at −80°C for 64 h revealed that the *Negativicutes* within *Firmicutes* were almost exclusively more abundant in the frozen samples, and no clear patterns at the phylum level were otherwise observed (Fig. S9).

### Distinct genera do not always exhibit the same abundance patterns across sample types.

**FIG 4:**
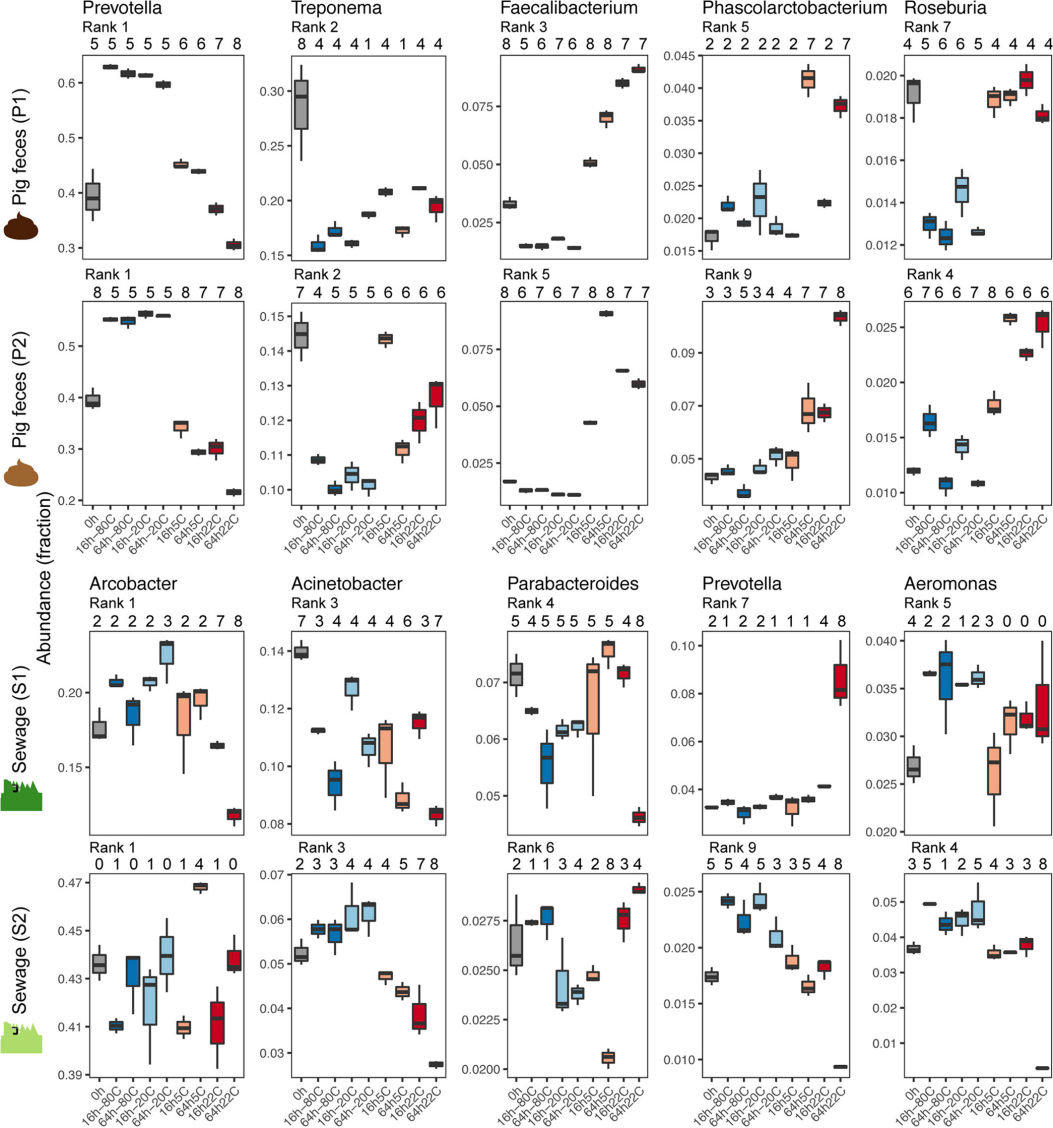
Box plots displaying the abundance of selected highly abundant bacteria in unspiked pig feces (P1 and P2) and sewage (S1 and S2) samples. The abundance rank is indicated for each genus in the specific sample. Differential abundance between the storage conditions was tested pairwise using DESeq2, and the number of times a storage condition was significantly different (*P* < 0.01) from another is indicated above the box plots.

To quantify the effect of storage conditions on microbial community composition, we tested for the differential abundance of genera in pairwise comparisons using DESeq analyses that did not take the compositionality of the data into account (Fig. 4; see Data Set S1 available from https://doi.org/10.6084/m9.figshare.12010983). *Prevotella* (class *Bacteroidia*), the most abundant genus in pig feces, was more abundant for both P1 and P2 in frozen samples than in samples undergoing immediate DNA isolation. At temperatures above freezing, *Prevotella* seemed to decrease in relation to temperature and time (i.e., *Prevotella* abundance decreased the higher the storage temperature and with the more time that passed [Fig. 4; see Data Set S1 available from https://doi.org/10.6084/m9.figshare.12010983). A decrease in the most abundant genus in pig feces might also explain the observed increase in evenness (Peilou’s evenness) in these samples (Fig. 1B). *Faecalibacterium* (class *Clostridia*) in pig feces exhibited the opposite pattern compared to *Prevotella* and was detected in both samples (P1 and P2) at a higher abundance with increasing temperature and time and at a lower abundance in frozen samples than what was detected in samples processed immediately. Treponema (class *Spirochaetes*) was observed at a higher abundance in the samples processed immediately following sample collection for both pig feces samples (P1 and P2) than in stored samples, independent of temperature and time. The opposite was observed for *Phascolarctobacterium* (class *Negativicutes*), for which a higher abundance was observed for stored samples than for samples processed immediately.

The two sewage samples (S1 and S2), in contrast to the pig feces samples, exhibited less concordant genus abundance patterns between each other, respectively, and to the pig feces samples (Fig. 4). However, as also observed for the two pig feces samples, *Prevotella* in sewage sample 2 was detected at the highest abundance in frozen samples compared to the other storage conditions (Fig. 4). Overall, some generalizable genera abundance patterns were observed. However, some differences were observed between samples from the same environment (e.g., S1 versus S2) and different environments (pig feces versus sewage), indicating that the effect of storage on specific genera is not entirely generalizable across samples from the same and different environments (Fig. 4; see Data Set S1 available from https://doi.org/10.6084/m9.figshare.12010983).

### Effect of storage conditions on the mock community.

Duplicate sets of pig feces and sewages samples were spiked with a mock community composed of 8 different microorganisms and were stored at the different temperatures for different periods of times (Fig. 1A; Tables S2 and S3). Overall, the abundance patterns of these microorganisms under the examined storage conditions appeared individualized for P1, P2, S1, and S2 with few exceptions (Fig. S10A). A generalizable effect was observed across all four samples for *Cryptosporidium*, resulting in higher relative abundances in samples that were frozen (−20°C and −80°C) than in samples that were processed immediately (0 h). The effect was most pronounced for the samples stored at −80°C (Fig. S10A). In addition, for all samples, a decrease in Staphylococcus and *Fusobacterium* abundances was observed compared to samples that were processed immediately (0 h). This effect was most pronounced for the sewage samples and was independent of storage temperature and time. Furthermore, an increase of *Saccharomyces* was observed for several samples, for example P1, S1, and S2 at 22°C, while a decrease was observed, for example, for S1 and S2 at both −80°C and −22°C (Fig. S10A). However, these taxa did not exhibit the same abundance patterns in spiked and unspiked samples even though the six bacterial genera were detected in many of the unspiked samples as part of the native microbial community (Fig. S10A and B). For example, in the unspiked samples, Salmonella in P2 exhibited a decrease in abundance under almost all storage conditions compared to samples that were processed immediately. This was not observed for the respective P2 samples spiked with additional cells of Salmonella added to the pig feces microbial community (Fig. S10A and B).

When the abundance of the mock community strains residing in the pig and sewage samples was analyzed in relative abundance to each other, it appeared that the abundance of Gram-negative bacteria was higher than Gram-positive bacteria and eukaryotes (*Cryptosporidium* and *Saccharomyces*) (Fig. S10C; Table S6). Nevertheless, we also observed a decrease of *Fusobacterium* and Staphylococcus abundances under certain storage conditions as described above (Fig. S10; Table S6). When the abundance of the mock community strains before spiking was analyzed based on cell counts and CFU, it appeared that the abundance of Gram-positive bacteria was higher than Gram-negative bacteria and eukaryotes (Fig. S10C; Table S6). When DNA from the individual mock community strains was isolated using the same DNA extraction protocol that was optimized and used for complex microbiome samples, more DNA was obtained from the Gram-negative bacteria than from Gram-positive bacteria and eukaryotes (Fig. S10C; Table S6). However, the concurrent analysis of both spiked and unspiked sets of samples throughout this study revealed that, overall, the same observations were obtained in regard to microbial community and antimicrobial resistome patterns for both sets of samples (e.g., compare Fig. 1B and Fig. S3, S4, S5, and S13), providing an additional layer of technical control in this study.

### Effect of freeze-thaw cycles on microbial community composition.

In order to assess the effect of repeated freeze-thaw cycles on inferred microbial community composition, separate aliquots of pig feces P1 and sewage S1 were frozen at −20°C and −80°C at 0 h, thawed and refrozen after 16 h, and thawed and frozen again after 40 h (2 freeze-thaw cycles), 64 h (3 freeze-thaw cycles), and 88 h (4 freeze-thaw cycles) (Fig. 1A; Table S1). We examined the inferred microbial community composition in PCoAs, and it appeared that the communities changed on a gradient according to an increasing number of freeze-thaw cycles, in particular for pig feces P1 (Fig. S11A). To reveal microorganisms that changed in abundance due to the repeated freeze-thaw cycles, CCAs were performed. For both pig feces and sewage, some eukaryotic genera as well as *Actinobacteria* and *Firmicutes* were more associated with samples undergoing increasing numbers of freeze-thaw cycles (Fig. S11B). Regarding eukaryotic genera, we, for example, observed in the spiked samples an increasing abundance of *Cryptosporidium* with increasing numbers of freeze-thaw cycles (Fig. S11C). In contrast, *Proteobacteria* and *Bacteroidetes* appeared to be more associated with low numbers of freeze-thaw cycles (Fig. S11B).

### A systematic effect on antimicrobial resistomes based on sample storage condition.

To examine whether antimicrobial resistance (AMR) patterns would change in response to the different storage conditions, we mapped the reads from all samples against the ResFinder database. A rarefaction analysis revealed that the majority of acquired AMR genes discoverable under these conditions were observed for the pig feces samples, as revealed through a flattening rarefaction curve (Fig. S12A). This was not the case for sewage samples that exhibited a higher AMR gene richness and lower total number of mapped reads than that observed in pig feces samples (Fig. S12A). Most AMR genes in pig feces represented genes conferring resistance to tetracycline (average abundance = 69.0%) and in sewage represented genes conferring resistance against macrolides (average abundance = 31.7%) (Fig. S12B). The second and third most abundant AMR classes in sewage represented AMR genes against tetracyclines (average abundance = 24.8%) and beta-lactams (average abundance = 23.6%) (Fig. S12B).

**FIG 5:**
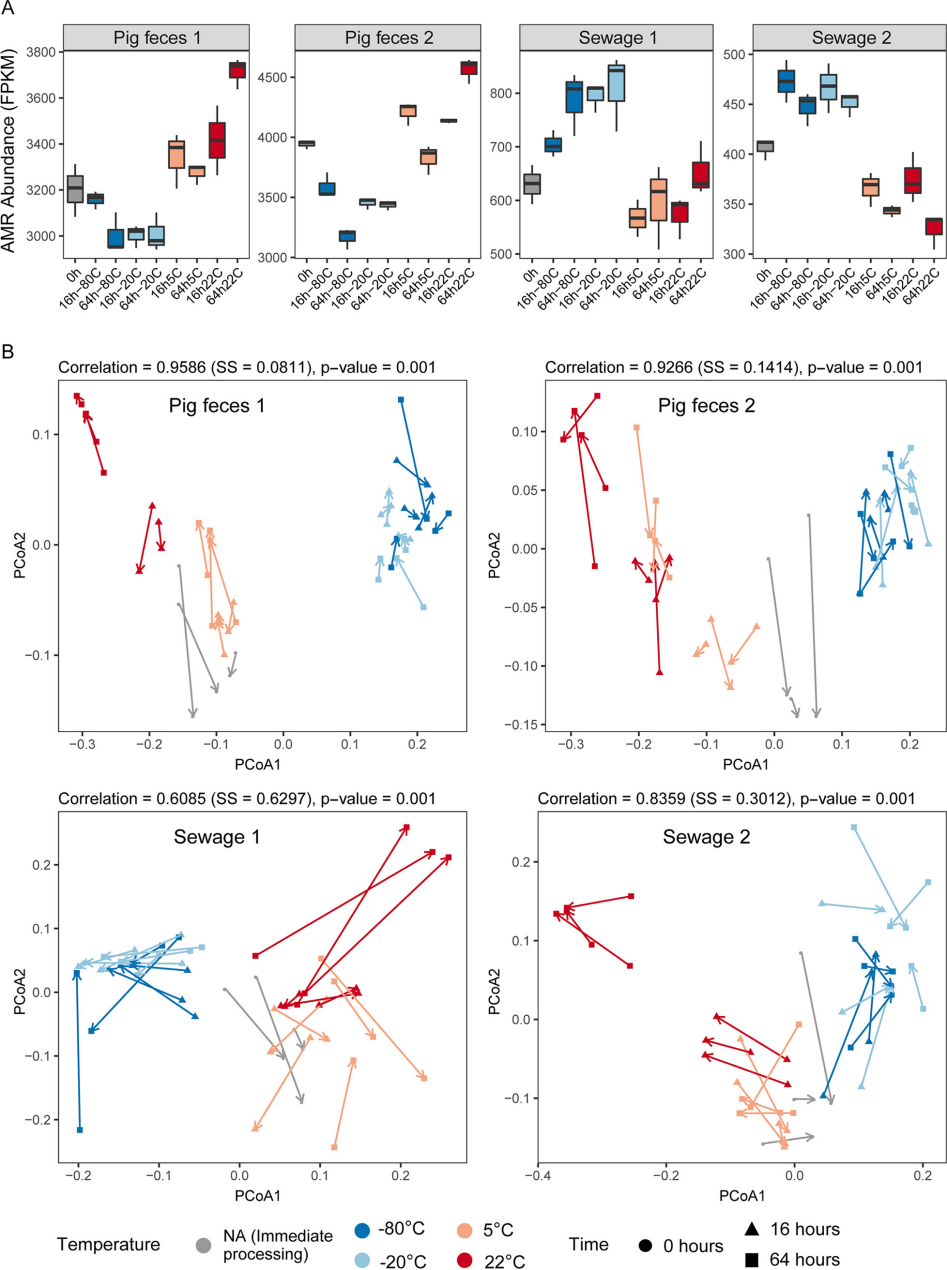
The effect of sample storage conditions on the resistome. (A) Box plots displaying total antimicrobial resistance (AMR) abundance in the unspiked samples (P1, P2, S1, and S2) measured in FPKM relative to the total number of bacterial reads. (B) Procrustes rotation comparing the resistome and taxonomic dissimilarities in the four unspiked samples. For the results of the spiked samples, see Fig. S13 in the supplemental material. SS, sum of squares.

Overall, the total observed abundance of AMR genes appeared to be dependent on the storage conditions, and opposite patterns were observed for pig feces and sewage samples (Fig. 5A; Fig. S13A). The total AMR abundance was lowest in pig feces (P1 and P2) when samples were frozen (−20°C and −80°C) and in sewage (S1 and S2) when stored at temperatures above freezing (22°C and 5°C). In contrast, total AMR abundance was highest in pig feces (S1 and S2) when stored at temperatures above freezing (22°C and 5°C) and in sewage (S1 and S2) when frozen (−20°C and −80°C) (Fig. 5A; Fig. S13A).

Comparing the resistome with taxonomic patterns using Procrustes analysis revealed that in all cases (P1, P2, S1 and S2) they correlated significantly (*P* < 0.001) (Fig. 5B; Fig. S13B). Storage had a systematic effect on the inferred taxonomic and antimicrobial resistome pattern, particularly the frozen samples that clustered together. Even though a clustering of samples stored at temperatures above 0°C was also observed, the resistome-based patterns were less pronounced than taxonomic-based patterns (Fig. 5B; Fig. S11B).

### AMR classes exhibit distinct abundance patterns under different storage conditions.

**FIG 6:**
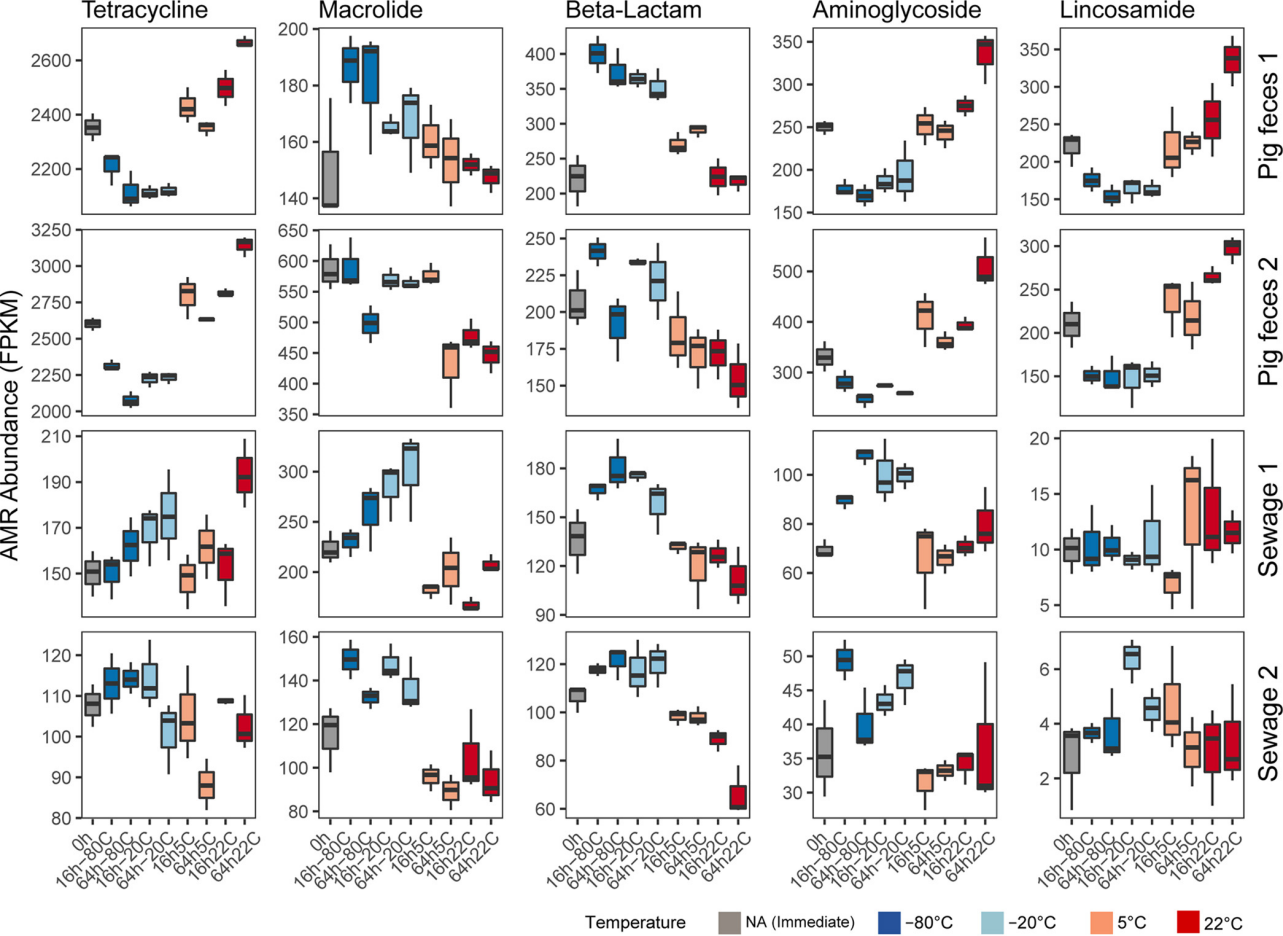
The effect of storage on the abundance of antimicrobial resistance classes. Box plots displaying total antimicrobial resistance (AMR) abundance for the most abundant antimicrobial resistance classes in the unspiked samples (P1, P2, S1, and S2). The abundance was measured in FPKM relative to the total number of bacterial reads. For the results of the spiked samples, see Fig. S14 in the supplemental material.

An analysis of the most abundant AMR classes revealed different effects of storage on the resistome across the sample types (pig feces and sewage). For pig feces (P1 and P2), tetracycline resistance genes were detected at a higher abundance in samples stored at temperatures above freezing (22°C and 5°C) and at a lower abundance in samples when frozen (−20°C and −80°C) than in samples that were processed immediately (Fig. 6; Fig. S14). A similar pattern was observed for aminoglycoside- and lincosamide-associated resistance genes. In contrast, macrolide- and beta-lactam-associated genes were detected at a higher abundance in samples stored frozen (−20°C and −80°C) than in samples processed immediately and stored at temperatures above freezing (22°C and 5°C) (Fig. 6; Fig. S14). In sewage (S1 and S2), the pattern for the top 4 most abundant AMR classes (macrolide, tetracycline, beta-lactam, and aminoglycoside) was similar in that, in most cases, AMR class abundance was highest when samples were stored frozen (−20°C and −80°C) (Fig. 6; Fig. S14). Macrolide and beta-lactam resistance gene abundance patterns were similar as observed in pig feces samples (i.e., AMR gene abundance was higher in frozen samples [−20°C and −80°C] than in samples stored at temperatures above 0°C [22°C and 5°C]).

## DISCUSSION

Metagenomics is a powerful technology to obtain insight about the taxonomic and functional composition of microbial communities. Standardization and harmonization of the laboratory procedures for metagenomics-based analyses are important, in particular for long-term studies that aim at investigating variations in microbial community composition over time; it would also facilitate meta-analyses involving data from different studies. Knowledge about the impact of different sample processing strategies would furthermore allow for the design of more precise algorithms for the identification and correction of batch effects. In general, it is critical to know what effect different sample processing steps have in skewing the true microbial community composition to facilitate improving laboratory protocols and more rigorous interpretations of metagenomics-based analyses.

Here, we focused on the impact of one of the key initial steps in metagenomics, namely, the storage conditions for biospecimens. This step can be difficult to standardize, and we chose to investigate different real-world scenarios. For example, regarding storage duration times, we investigated immediate sample processing (0 h) as well as storage for 16 h and 64 h, resembling situations where samples are received in the afternoon and sample processing can be first initiated the following day or after a weekend, respectively. We also aimed at examining situations where samples have to be stored for extended periods of time (4, 8, and 12 months). Storage temperatures were chosen to reflect common ways to store samples, such as in the refrigerator (5°C), freezer (−20°C), and deep freezer (−80°C), but also storage temperatures that might be experienced where cooling and freezing are not an option (e.g., 22°C), such as in field studies. In addition, we examined the effect of freeze-thaw cycles, a scenario where one has to repeatedly retrieve aliquots from the biospecimen stored in the freezer, for example, for additional rounds of DNA isolation or other types of analysis such as metabolomics, and where not the entire specimen is kept in the frozen state.

Overall, we found that the four investigated biospecimens could still be distinguished, independent of the effect introduced by the different storage conditions, as they clustered by sample origin and not based on storage condition. This is in support of previous findings (11). However, we anticipate that it would not necessarily always be possible to trace back samples to their origin using standard metagenomics analyses if one is examining a larger set of samples of the same type (e.g., pig feces). Therefore, it is advised to store all samples in the same way and ideally under frozen conditions (−20°C or −80°C), under which we observed the fewest changes in microbial community composition over time.

We found that storage had a systematic effect on the inferred taxonomical microbiome composition. For example, frozen samples (–20°C or –80°C) clustered together and were more similar to each other than samples stored at temperatures above freezing (5°C and 22°C). It should be noted that while freezing the samples changed the detected microbiome compared to immediately processing the samples, the results were stable over time. Previous 16S rRNA-based studies on human feces have found different results regarding the effect of different sample storage conditions on alpha and beta diversity as well as taxa abundances changes (13, 15, 16).

We also observed that storage conditions had a systematic effect on the antimicrobial resistome that correlated with the taxonomic analysis. For all samples stored at temperatures above freezing (5°C and 22°C), we observed a higher abundance of taxa related to the phyla *Firmicutes* and *Actinobacteria* and a lower abundance of taxa related to *Bacteroidetes* and *Proteobacteria*, which were more associated with the frozen samples (−80°C and −20°C). Of note, while the most abundant genera exhibited abundance changes similarly in both pig feces samples (e.g., *Prevotella*, Treponema, and *Faecalibacterium*), this was not the case for the two sewage samples. This could be impacted by a higher degree of differences in chemical composition, physical properties, and water content in the sewage samples than in the pig feces samples (2, 17–19). This may also be supported by the observation that both alpha and beta diversity were more similar between the two pig feces samples than between the two sewage samples, respectively.

We spiked a subset of all samples with a well-characterized mock community and examined abundance changes of these in response to the different storage conditions. Interestingly, the members of this mock community did not seem to respond to the storage conditions in a manner similar to the close relatives of the native community, even though the six bacterial genera were also present in many of the unspiked samples as part of the native microbial community. We cultivated the mock community members on plates in the laboratory. Hence, they might not exhibit the same physiological state as the native members, not be able to rapidly accommodate to the new conditions, and respond differently to the DNA isolation procedure (2). Differences between the theoretical and observed abundances of spiked-in bacteria have been previously observed (20–24). In contrast to bacteria and fungi, *Cryptosporidium* oocysts are likely to behave similar in this setting as in natural settings due to the dormant nature of this stage. The findings of an increase in *Cryptosporidium* under freezing conditions are therefore of relevance. It should, however, be mentioned that under clinical settings, a sample may additionally contain other stages of *Cryptosporidium* for which improved lysis might not require freezing.

In the subexperiment involving repeated freeze-thaw cycles, we observed an increase in the abundance of eukaryotes. For studies aimed at detecting microbial eukaryotes, freezing the biospecimens seems, therefore, to have a positive impact on sensitivity. For instance, the clinically relevant Cryptosporidium parvum that was part of the mock community had a relatively higher abundance in samples that underwent 3 to 4 freeze-thaw cycles than in samples that underwent 2 freeze-thaw cycles. Furthermore, an increasing abundance of *Actinobacteria* and *Firmicutes* was observed upon increasing the number of freeze-thaw cycles, suggesting that this procedure facilitated a disruption of more rigid cell walls. However, repeated freeze-thaw cycles may also contribute to a degradation of DNA that is more easily released from Gram-negative bacteria, contributing to a decreasing abundance observed for these taxa. This is supported by the finding that a higher abundance of *Bacteroides* and *Proteobacteria* was more associated with samples undergoing 2 freeze-thaw cycles than in samples undergoing 3 to 4 freeze-thaw cycles.

Overall, we recommend that all samples be stored in the freezer (−20°C or −80°C) before DNA isolation. While microbial community composition remained stable under these conditions over shorter periods of time, it remains to be investigated more thoroughly whether this would also be the case for extended periods of time, as samples within a study might be stored for different lengths of time. The strategy of storing samples in the freezer could be an advantage as generally most samples are stored frozen, facilitating better comparability between studies. If it is not possible to store samples in the freezer, it would be good to perform DNA isolation immediately following arrival at the lab if feasible for all samples in a study. If this is not possible, we recommend storing samples in the refrigerator and processing them the same day or the following day for all samples. It could also be possible to store samples using preservation reagents (12, 13, 25); however, these interventions might interfere with other investigations that will also be conducted on the same biospecimen (e.g., metabolomics, proteomics, transcriptomics, and cultivation). Within a project, standardization of storage conditions is key for generating comparable data. We strongly recommend that more details regarding sample storage and processing conditions be provided as part of the metadata submitted together with the DNA sequencing data to public repositories than is currently provided (26).

## MATERIALS AND METHODS

### Microbiome samples.

Two pig feces and two domestic sewage samples were collected for this study. The pig feces samples were obtained from two individual animals right after defecation at two different conventional pig production farms in Denmark on separate occasions and were transported within 3 h to the laboratory in a cooling box. The unprocessed domestic sewage was collected at a local wastewater treatment facility (Lyngby-Taarbaek Forsyningen) on separate occasions as 20-liter inlet water in 5-liter sterilized plastic bottles, transported in cooling boxes to the laboratory within 20 min, and placed into a refrigerator at 5°C. Sedimentation of the sewage was commenced immediately using 50-ml Falcon tubes and two centrifuges (Eppendorf 5810R, Hamburg, Germany; program: 10 min at 10,000 × *g*). Sedimentation of the entire sewage sample was completed within 12 h.

Each individual pig feces sample and sewage pellet was thoroughly homogenized in a 50-ml Falcon tube with a sterile wooden spatula and distributed into two large aliquots in 50-ml Falcon tubes, respectively. One aliquot for each of the four original microbiome samples, respectively, was spiked with a freshly prepared mock community (see below), and all samples were homogenized with a sterile wooden spatula to take into account that the mixing might have an effect on community composition. Small aliquots for each sample storage condition were prepared in Eppendorf tubes and either processed immediately (storage for 0 h) or stored at a certain temperature (−80°C, −20°C, 5°C, or 22°C) for a specific period of time (16 h, 64 h, 4 months, 8 months, or 12 months) (described in detail below). Additional aliquots underwent 2 to 4 freeze-thaw cycles at different time points (40 h, 64 h, or 88 h) (described in detail below).

### Mock community.

In parallel to microbiome sample collection and initial processing, a mock community was prepared at the four individual occasions. The mock community consisted of eight different microorganisms: Propionibacterium freudenreichii DSM20271, Bacteroides fragilis NCTC9343, Staphylococcus aureus NCTC8325, Fusobacterium nucleatum ATCC 25586, Escherichia coli ATCC 25922, Salmonella enterica ATCC 14028S, Cryptosporidium parvum Iowa II isolate, and Saccharomyces cerevisiae S288C, representing two different domains of life (*Bacteria* and *Eukarya*) and seven phyla. The bacterial domain was represented by five phyla (*Actinobacteria*, *Bacteroidetes*, *Firmicutes*, *Fusobacteria*, and *Proteobacteria*), which differed in terms of cellular morphology (cocci, rods, and fusiform rods), cell wall structure (Gram-positive and Gram-negative), as well as oxygen requirements. The eukaryotic domain was represented by two different phyla (*Apicomplexa* and *Ascomycota*). The specific microorganisms were chosen because their whole-genome sequence was publicly available. Additional information about the mock community microorganisms, including cultivation conditions, is provided in Table S2 in the supplemental material. The microorganisms were transferred to an Eppendorf tube (1.5 ml) with an inoculation loop and resuspended in sterile phosphate-buffered saline (PBS; Gibco, Paisley, UK) with a table-top vortex mixer. Enumeration of bacteria and fungi was performed using a Petroff counting chamber under a light microscope, counting two diagonal corners on two separately prepared slides. Organisms cultured aerobically (St. aureus, E. coli, Sl. enterica, and Sc. cerevisiae) were processed first. Raw cell counts and volumes of resuspended cells that were used for the spiking of fecal and sewage samples are provided in Table S3. The C. parvum oocysts were obtained from Waterborne Inc. in PBS, amounting to 1.2 × 10^8^ cells, as determined by fluorescence-activated cell sorter (FACS) by the provider. The oocysts were washed 5 times by resuspending them in 10 ml of PBS and sedimenting them using a centrifuge (Eppendorf 5810R, Hamburg Germany; 1,000 × *g* for 10 min.). The mock community was mixed, leading to 10^9^ cells/mg (for P1, 5 × 10^8^) Gram-positive bacteria, 10^8^ cells/mg Gram-negative bacteria, 2 × 10^7^ cells/mg Sc. cerevisiae, and 2 × 10^6^ cells/mg C. parvum in pig feces and pelleted sewage samples, respectively (Table S3). CFU counts were subsequently obtained to estimate the number of live cells. CFU values were determined for selected organisms and were dependent on the microbiome sample that was processed (Table S3).

### Sample storage conditions.

The effect of different sample storage conditions was investigated at four temperatures (deep freezing, −80°C; freezing, −20°C; refrigerator, 5°C; room temperature, 22°C) and six storage duration times representing relevant situations in microbiome studies (immediate DNA isolation, 0 h; sample storage overnight, 16 h; sample storage over a weekend, 64 h; longer-term sample storage, 4 months, 8 months, and 12 months). To assess different sample matrices and different samples of the same matrix, two pig feces samples (P1 and P2) and two sewage samples (S1 and S2) were included (for long-term storage, only P1 and S1 samples were used). The technical variability was accounted for by processing all subsamples in triplicate from the DNA isolation step.

In addition, given that, in microbiome studies, sample aliquots may be repeatedly retrieved from the main sample stored in a freezer, we aimed at simulating this in a subexperiment by performing a series of freeze-thaw cycles. Aliquots were placed in the freezer (−20°C) and deep freezer (−80°C) and were thawed and refrozen after 40 h (2 freeze-thaw cycles), 64 h (3 freeze-thaw cycles), and 88 h (4 freeze-thaw cycles). An overview of all 343 samples, including controls, is provided in Table S1.

### DNA isolation and metagenomics shotgun sequencing.

DNA isolation was performed according to a modified QIAamp Fast DNA stool minikit (Qiagen) protocol that included a bead beating step with MoBio garnet beads and additional steps optimized for pig feces and sewage (https://doi.org/10.6084/m9.figshare.3475406) (2). Using the same DNA isolation procedure, DNA was also isolated from aliquots of each individual strain of the mock community. A DNA isolation blank control was included at each round of DNA isolation. Positive controls consisted of the pure mock community, prepared at the four individual occasions, and they were processed in triplicate together with the other samples at the 0-h time points, respectively. DNA concentrations were measured with a Qubit dsDNA high sensitivity (HS) assay kit on a Qubit 2.0 fluorometer (Invitrogen, Carlsbad, CA) before storing the DNA at –20°C.

Metagenomics shotgun sequencing was for, the majority of samples (i.e., 287 samples), performed on an Illumina HiSeq 4000 (2 × 150 cycles, paired end [PE]) at the Oklahoma Medical Research Foundation (USA). DNA was processed for sequencing, involving mechanical fragmentation (Covaris E220evolution; aimed insert size = 350 bp), followed by amplification-free library preparation using a NEXTflex PCR Free DNA prep kit (Bioo Scientific) according to standard manufacturer protocols. The remaining samples (i.e., 56 samples) associated with long-term storage (4, 8, and 12 months) of P1 and S1 were sequenced on an Illumina HiSeq 4000 (2 × 150 cycles, paired end) at Admera Health, New Jersey (USA). DNA was processed for sequencing, involving mechanical fragmentation (Covaris E220evolution; aimed insert size = 350 bp), followed by amplification-free library preparation using a KAPA library preparation kit (Roche) according to standard manufacturer protocols. Samples were distributed randomly across flow cell lanes. Labeling issues occurred for four samples, resulting in the swapping of these samples. It was possible to backtrack the samples that were mislabeled based on lab book entries, DNA concentration measurements performed at both the research laboratory and sequencing provider, and by using principal-coordinates analysis of Bray-Curtis distances performed corresponding to the PCoA analysis described in detail below. These four samples, of which one was a DNA isolation blank control, were excluded from further analysis to ensure mislabeling did not have an effect on the interpretation of results. Furthermore, one sample was excluded due to low sequencing output.

### Sequencing data analysis.

Demultiplexed raw reads were processed with BBduk2 as part of the BBTools package (http://jgi.doe.gov/data-and-tools/bbtools/) to remove low-quality bases from reads (trimq = 20), reads shorter than 50 bp, and adapter sequences. Mapping of processed paired-end reads was performed with MGmapper (27) that incorporates a Burrows-Wheeler aligner (BWA-mem). Reads were mapped in bestmode against 11 databases (human, bacteria, bacteria_draft, fungi, archaea, virus, parasites_vertebrates, parasites_other, HumanMicrobiome, common_animals, and common_plants) containing genome sequences that were downloaded from NCBI (Table S7) with a 0.8 fraction of matches+mismatches relative to the full length of the read (27). Details about the number of raw reads (average, 14.4 million PE reads; range, 2.9 to 41.0 million PE reads per sample), number of reads that passed the quality filtering (average, 12.4 million PE reads; range, 2.5 to 35.9 million PE reads), and mapped reads (average, 2.2 million PE reads; range, 0.65 to 7.1 million PE reads) are provided in Table S1. Genome assembly sizes were used for normalization according to genome size. In addition, increased hit counts to specific contigs were adjusted as implemented previously (6).

The taxonomic-based analyses were performed with reads mapping to genomes of all bacteria, archaea, viruses, fungi, and *Cryptosporidium*, and analyses were performed by aggregating counts to the level of genera. The mapping to functional databases (ResFinder, Virulencefactors, Virulence) was performed in “fullmode” (allowing reads to be assigned to multiple databases), and the same mapping criteria as described above had to be fulfilled. ResFinder was the only database that was used for further analysis and includes acquired resistance genes and chromosomal point mutations (28). Resistance genes were aggregated with respect to resistance against different antimicrobial classes.

### Statistical analysis.

*Preprocessing.* The read count table was first processed by dividing all counts by two, since mapping was performed as proper pairs (mapping of a forward and reverse read); this is referred to as the raw count table (see item A at https://doi.org/10.6084/m9.figshare.12010989). The raw count table was normalized according to genome size for each microorganism, followed by total sum scaling. The resulting table is referred to as the count table (see items B and C at https://doi.org/10.6084/m9.figshare.12010989). The microorganisms listed in the tables were aggregated to genus level. For the antimicrobial resistome analysis, raw read counts are provided for all genes divided by two (see item D at https://doi.org/10.6084/m9.figshare.12010989) and normalized according to the total bacterial reads per sample (mapping to the databases bacteria, bacteria_draft, and HumanMicrobiome) as fragments per kilobase reference per million bacterial fragments (FPKM) (see item E at https://doi.org/10.6084/m9.figshare.12010989). The FPKM table was also aggregated to resistance against antimicrobial classes (see item F at https://doi.org/10.6084/m9.figshare.12010989), and the class-level information for each AMR gene is available as item G at https://doi.org/10.6084/m9.figshare.12010989.

All statistical analyses and visualization of data were performed in R (version 3.4.4, R Foundation for Statistical Computing, Vienna, Austria) (29). The multivariate community ecology analysis was performed with vegan version 2.5.2 (30), and the differential abundance analysis was performed with DESeq2 version 1.18.1 (31). Visualization of data was performed with ggplot2 version 2.3.0 (32) and pheatmap version 1.0.10. All analyses were performed separately for both spiked and unspiked samples. Final modifications to figures were made in Inkscape or Illustrator.

A high degree of concordance was observed between the unspiked and spiked samples. The majority of data presented in the main text are based on the unspiked samples. The same analyses of the spiked samples are presented in the supplemental material.

*Alpha diversity*. Alpha diversity was calculated based on the raw count table estimating richness (Chao1), evenness (Pielou’s), and diversity (Simpson) using the diversity function in vegan. Box plots were created for each sample and according to the different storage conditions.

*Dissimilarity calculations*. A dissimilarity matrix was generated based on Bray-Curtis dissimilarities on a Hellinger transformed count table, resulting in pairwise dissimilarity values between all of the individual samples. This is referred to as the dissimilarity object, which was also used in the adonis analysis, principal-coordinates analyses (PCoA), and Procrustes analysis. Statistical tests comparing dissimilarities were performed between the different groups of samples (i.e., within replicates, within the same sample matrix, and between the different sample matrices). A Levene’s test on raw dissimilarities between groups and log-transformed dissimilarities revealed that a nonparametric test was appropriate. A Kruskal-Wallis test was used to test whether there was an overall difference between groups, and, if significant (*P* < 0.05), a *post hoc* analysis was performed using a Dunn pairwise test of groups as part of the FSA package version 0.8.20.

*Heat maps*. To visualize the taxonomic and resistance gene abundance, heat maps were generated using the pheatmap package. In the heat map visualization, microbial genera were standardized to zero mean and unit variance, including the 30 most abundant genera. Sample-based dendrograms were generated from the dissimilarity object using complete-linkage clustering. The microorganism-based dendrograms were generated using Pearson product-moment correlation coefficients with complete-linkage clustering on the dissimilarity object.

*Adonis*. The “adonis2” function as part of the vegan package was used to perform an analysis of variance. The function takes a dissimilarity object and partitions dissimilarities for the sources of variation. It is a permutational approach, and, in doing so, the significances of the partitions can be assessed. The “betadisper” function was used to assess the differences in group homogeneities, and the fitted models were analyzed with “permutest”. If significant (*P* < 0.05), the model was considered inappropriate for the analysis.

*Principal-coordinate analysis*. PCoA plots were generated using the “capscale” function unconstrained as part of the vegan package. The inbuilt stressplot function in vegan was used to extract the ordination distance and corresponding dissimilarities to make stress plots in ggplot2. The coordinates and eigenvalues were extracted from the capscale object to create PCoA plots and scree plots and visualized with ggplot2. How well the PCoA performed at visualizing the data was evaluated in the stress plots. The scree plots were used to evaluate how much variance was explained on the different axes.

*Procrustes*. Procrustes rotation was performed with the vegan function “protest” to compare the principal coordinates of the taxonomy-based dissimilarities and the antimicrobial resistome-based dissimilarities using classical multidimensional scaling (cmdscale). The descriptive statistics were extracted from the object generated from ‘protest’ and included in the plot made with ggplot2.

*Canonical correspondence analysis*. A constrained visualization of the data was performed with the “cca” function in vegan on the raw count table. CCA is based on the chi-square distance, and, therefore, the raw count table was used. The cca plot, scree plots, and stress plots were generated from the cca object using ggplot2. The genera in the plot were colored according to different selected taxonomical groups or antimicrobial resistance (AMR) classes based on occurrence and abundance in the data set.

*Differential abundance analysis*. Differential abundance analyses were performed using DESeq (31) at sample matrix level (P1, P2, S1, and S2) using the raw count table. Custom DESeq sizeFactors were used, taking the total sums for the different samples divided by the mean total sums for all of the samples. Pairwise comparisons of the abundance of the genera were performed between storage conditions using DESeq2. Based on the PCoA plots and raw dissimilarities, the differences between 0 h and 64 h (–80°C), 0 h and 64 h (22°C), and 0 h and 64 h (5°C) were further assessed through the use of MA plots with the genera colored according to the same criteria as in the CCA (see above). Comparison of the same genera or AMR genes across the different samples was performed by extracting log_2_ fold change and generating box plots to compare across all samples and scatter plots to compare the two pig feces and sewage samples separately.

*Mock community*. Line plots were generated by relativizing to the mean at 0 h for spiked and unspiked samples, respectively. All values were log_2_ scaled, and the 95% confidence interval for each measurement is represented in the plots. Stacked bar charts for each storage condition and sample were created for the detected mock community organisms and compared with microscopy-based cell counts, CFU, and concentrations from the individual DNA isolations obtained for the individual mock community members used for assembling the mock community. To account for the background in the spiked samples, a factor (spiked/unspiked) was calculated from the nonmock community genera present in the samples. This factor was unique for sample and storage condition. The factor was used to multiply mock community genera in the unspiked samples to obtain a more precise estimate of the background in the spiked samples and then subtracted from the respective genera in the spiked samples. This procedure was performed to account for the compositionality of data and that the mock community recruits reads from the background.

### Data availability.

The raw reads are accessible from the International Nucleotide Sequence Database Collaboration (INSDC) at ENA/EBI, DDBJ, and NCBI under project accession PRJEB31650. Additional data are available from Figshare at https://figshare.com/projects/Standard_sample_storage_conditions_impact_on_inferred_microbiome_composition_and_antimicrobial_resistance_patterns/76044.

## References

[1] Jones MB, Highlander SK, Anderson EL, Li W, Dayrit M, Klitgord N, Fabani MM, Seguritan V, Green J, Pride DT, Yooseph S, Biggs W, Nelson KE, Venter JC. 2015. Library preparation methodology can influence genomic and functional predictions in human microbiome research. Proc Natl Acad Sci USA 112:14024–14029. doi:10.1073/pnas.1519288112. 26512100 PMC4653211

[2] Knudsen BE, Bergmark L, Munk P, Lukjancenko O, Prieme A, Aarestrup FM, Pamp SJ. 2016. Impact of sample type and DNA isolation procedure on genomic inference of microbiome composition. mSystems 1:e00095-16. doi:10.1128/mSystems.00095-16. 27822556 PMC5080404

[3] Peabody MA, Van Rossum T, Lo R, Brinkman FSL. 2015. Evaluation of shotgun metagenomics sequence classification methods using *in silico* and *in vitro* simulated communities. BMC Bioinformatics 16:363. doi:10.1186/s12859-015-0788-5. PMC4634789 26537885

[4] Bowers RM, Clum A, Tice H, Lim J, Singh K, Ciobanu D, Ngan CY, Cheng J-F, Tringe SG, Woyke T. 2015. Impact of library preparation protocols and template quantity on the metagenomic reconstruction of a mock microbial community. BMC Genomics 16:856. doi:10.1186/s12864-015-2063-6. 26496746 PMC4619416

[5] Wiehlmann L, Pienkowska K, Hedtfeld S, Dorda M, Tümmler B. 2017. Impact of sample processing on human airways microbial metagenomes. J Biotechnol 250:51–55. doi:10.1016/j.jbiotec.2017.01.001. 28119120

[6] Hendriksen RS, Munk P, Njage P, Bunnik B, McNally L, Lukjancenko O, Röder T, Nieuwenhuijse D, Pedersen SK, Kjeldgaard J, Kaas RS, Clausen PTLC, Vogt JK, Leekitcharoenphon P, Schans MGM, Zuidema T, Husman AMR, Rasmussen S, Petersen B, Global Sewage Surveillance project consortium, Amid C, Cochrane G, Sicheritz-Ponten T, Schmitt H, Alvarez JRM, Aidara-Kane A, Pamp SJ, Lund O, Hald T, Woolhouse M, Koopmans MP, Vigre H, Petersen TN, Aarestrup FM. 2019. Global monitoring of antimicrobial resistance based on metagenomics analyses of urban sewage. Nat Commun 10:1124. doi:10.1038/s41467-019-08853-3. 30850636 PMC6408512

[7] Aarestrup FM, Woolhouse MEJ. 2020. Using sewage for surveillance of antimicrobial resistance. Science 367:630–632. doi:10.1126/science.aba3432. 32029617

[8] Vogtmann E, Chen J, Kibriya MG, Chen Y, Islam T, Eunes M, Ahmed A, Naher J, Rahman A, Amir A, Shi J, Abnet CC, Nelson H, Knight R, Chia N, Ahsan H, Sinha R. 2017. Comparison of fecal collection methods for microbiota studies in Bangladesh. Appl Environ Microbiol 83:e00361-17. doi:10.1128/AEM.00361-17. 28258145 PMC5411505

[9] Flores R, Shi J, Yu G, Ma B, Ravel J, Goedert JJ, Sinha R. 2015. Collection media and delayed freezing effects on microbial composition of human stool. Microbiome 3:33. doi:10.1186/s40168-015-0092-7. 26269741 PMC4534027

[10] Dominianni C, Wu J, Hayes RB, Ahn J. 2014. Comparison of methods for fecal microbiome biospecimen collection. BMC Microbiol 14:103. doi:10.1186/1471-2180-14-103. 24758293 PMC4005852

[11] Blekhman R, Tang K, Archie EA, Barreiro LB, Johnson ZP, Wilson ME, Kohn J, Yuan ML, Gesquiere L, Grieneisen LE, Tung J. 2016. Common methods for fecal sample storage in field studies yield consistent signatures of individual identity in microbiome sequencing data. Sci Rep 6:31519. doi:10.1038/srep31519. 27528013 PMC4985740

[12] Choo JM, Leong LEX, Rogers GB. 2015. Sample storage conditions significantly influence faecal microbiome profiles. Sci Rep 5:16350. doi:10.1038/srep16350. 26572876 PMC4648095

[13] Panek M, Paljetak HC, Baresic A, Peric M, Matijasic M, Lojkic I, Bender DV, Krznaric Z, Verbanac D. 2018. Methodology challenges in studying human gut microbiota—effects of collection, storage, DNA extraction and next generation sequencing technologies. Sci Rep 8:5143. doi:10.1038/s41598-018-23296-4. 29572539 PMC5865204

[14] Poulsen CS, Pamp SJ, Ekstrøm CT, Aarestrup FM. Library preparation and sequencing platform introduce bias in metagenomics characterisation of microbial communities. 2019. bioRxiv. 10.1101/592154. PMC9045301 35289669

[15] Tedjo DI, Jonkers DMAE, Savelkoul PH, Masclee AA, van Best N, Pierik MJ, Penders J. 2015. The effect of sampling and storage on the fecal microbiota composition in healthy and diseased subjects. PLoS One 10:e0126685. doi:10.1371/journal.pone.0126685. 26024217 PMC4449036

[16] Bassis CM, Moore NM, Lolans K, Seekatz AM, Weinstein RA, Young VB, Hayden MK, for the CDC Prevention Epicenters Program. 2017. Comparison of stool versus rectal swab samples and storage conditions on bacterial community profiles. BMC Microbiol 17:78. doi:10.1186/s12866-017-0983-9. 28359329 PMC5374586

[17] Roager HM, Hansen LBS, Bahl MI, Frandsen HL, Carvalho V, Gøbel RJ, Dalgaard MD, Plichta DR, Sparholt MH, Vestergaard H, Hansen T, Sicheritz-Ponten T, Nielsen HB, Pedersen O, Lauritzen L, Kristensen M, Gupta R, Licht TR. 2016. Colonic transit time is related to bacterial metabolism and mucosal turnover in the gut. Nat Microbiol 1:16093. doi:10.1038/nmicrobiol.2016.93. 27562254

[18] Vandeputte D, Falony G, Vieira-Silva S, Tito RY, Joossens M, Raes J. 2016. Stool consistency is strongly associated with gut microbiota richness and composition, enterotypes and bacterial growth rates. Gut 65:57–62. doi:10.1136/gutjnl-2015-309618. 26069274 PMC4717365

[19] Falony G, Joossens M, Vieira-Silva S, Wang J, Darzi Y, Faust K, Kurilshikov A, Bonder MJ, Valles-Colomer M, Vandeputte D, Tito RY, Chaffron S, Rymenans L, Verspecht C, De Sutter L, Lima-Mendez G, D'hoe K, Jonckheere K, Homola D, Garcia R, Tigchelaar EF, Eeckhaudt L, Fu J, Henckaerts L, Zhernakova A, Wijmenga C, Raes J. 2016. Population-level analysis of gut microbiome variation. Science 352:560–564. doi:10.1126/science.aad3503. 27126039

[20] Hallmaier-Wacker LK, Lueert S, Roos C, Knauf S. 2018. The impact of storage buffer, DNA extraction method, and polymerase on microbial analysis. Sci Rep 8:6292. doi:10.1038/s41598-018-24573-y. 29674641 PMC5908915

[21] Hang J, Desai V, Zavaljevski N, Yang Y, Lin X, Satya R, Martinez LJ, Blaylock JM, Jarman RG, Thomas SJ, Kuschner RA. 2014. 16S rRNA gene pyrosequencing of reference and clinical samples and investigation of the temperature stability of microbiome profiles. Microbiome 2:31. doi:10.1186/2049-2618-2-31. 25228989 PMC4165438

[22] Ducarmon QR, Hornung BVH, Geelen AR, Kuijper EJ, Zwittink RD. 2020. Toward standards in clinical microbiota studies: comparison of three DNA extraction methods and two bioinformatic pipelines. mSystems 5:e00547-19. doi:10.1128/mSystems.00547-19. 32047058 PMC7018525

[23] Rausch P, Rühlemann M, Hermes BM, Doms S, Dagan T, Dierking K, Domin H, Fraune S, Frieling J, Hentschel U, Heinsen F-A, Höppner M, Jahn MT, Jaspers C, Kissoyan KAB, Langfeldt D, Rehman A, Reusch TBH, Roeder T, Schmitz RA, Schulenburg H, Soluch R, Sommer F, Stukenbrock E, Weiland-Bräuer N, Rosenstiel P, Franke A, Bosch T, Baines JF. 2019. Comparative analysis of amplicon and metagenomic sequencing methods reveals key features in the evolution of animal metaorganisms. Microbiome 7:133. doi:10.1186/s40168-019-0743-1. 31521200 PMC6744666

[24] Jovel J, Patterson J, Wang W, Hotte N, O'Keefe S, Mitchel T, Perry T, Kao D, Mason AL, Madsen KL, Wong GKS. 2016. Characterization of the gut microbiome using 16S or shotgun metagenomics. Front Microbiol 7:459. doi:10.3389/fmicb.2016.00459. 27148170 PMC4837688

[25] Song SJ, Amir A, Metcalf JL, Amato KR, Xu ZZ, Humphrey G, Knight R. 2016. Preservation methods differ in fecal microbiome stability, affecting suitability for field studies. mSystems 1:e00021-16. 10.1128/mSystems.00021-16 PMC5069758 27822526

[26] Kottmann R, Gray T, Murphy S, Kagan L, Kravitz S, Lombardot T, Field D, Glöckner FO, Genomic Standards Consortium. 2008. A standard MIGS/MIMS compliant XML schema: toward the development of the genomic contextual data markup language (GCDML). OMICS 12:115–121. doi:10.1089/omi.2008.0A10. 18479204

[27] Petersen TN, Lukjancenko O, Thomsen MCF, Sperotto MM, Lund O, Møller Aarestrup F, Sicheritz-Ponten T. 2017. MGmapper: reference based mapping and taxonomy annotation of metagenomics sequence reads. PLoS One 12:e0176469. doi:10.1371/journal.pone.0176469. 28467460 PMC5415185

[28] Zankari E, Hasman H, Cosentino S, Vestergaard M, Rasmussen S, Lund O, Aarestrup FM, Larsen MV. 2012. Identification of acquired antimicrobial resistance genes. J Antimicrob Chemother 67:2640–2644. doi:10.1093/jac/dks261. 22782487 PMC3468078

[29] R Core Team. 2017. R: a language and environment for statistical computing. R Foundation for Statistical Computing, Vienna, Austria.

[30] Oksanen J, Blanchet FG, Friendly M, Kindt R, Legendre P, McGlinn D, Minchin PR, O’Hara RB, Simpson GL, Solymos P, Stevens MHH, Szoecs E, Wagner H. 2007. The vegan package. Community ecology package. https://github.com/vegandevs/vegan.

[31] Anders S, Huber W. 2010. Differential expression analysis for sequence count data. Genome Biol 11:R106. doi:10.1186/gb-2010-11-10-r106. 20979621 PMC3218662

[32] Wickham H. 2016. ggplot2: elegant graphics for data analysis. Springer Verlag, New York, NY.

