# Supplementary material for: Standard Sample Storage Conditions Have an Impact on Inferred Microbiome Composition and Antimicrobial Resistance Patterns
Source: Microbiol Spectr. 2021 Oct 6;9(2):e01387-21. doi: 10.1128/Spectrum.01387-21 (PMC8510183; doi:10.1128/Spectrum.01387-21)
Supplement: SUPPLEMENTAL FILE 8 — Supplemental material. Download Spectrum.01387-21-s0008.pdf, PDF file, 5.5 MB [file spectrum.01387-21-s0008.pdf]

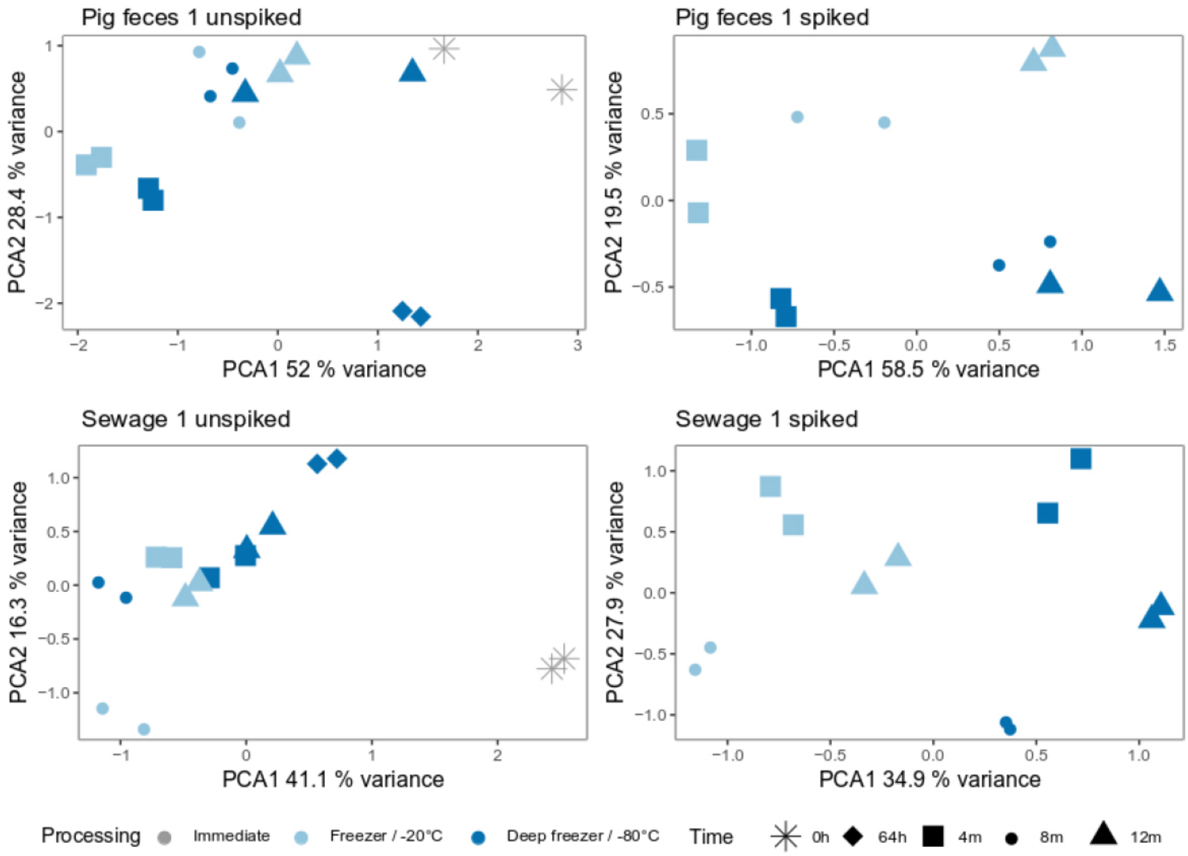

Figure S1: The effect of long-term storage. Principal component analysis (PCA), for pig feces 1 (unspiked/spiked with mock community) and sewage 1 (unspiked/spiked with mock community), to explore the effect of freeze-thaw cycles. PCA were generated from an Euclidean distance matrix obtained from isometric log-ratio transformed data. Variance explained by the first two dimensions are indicated at the axes.

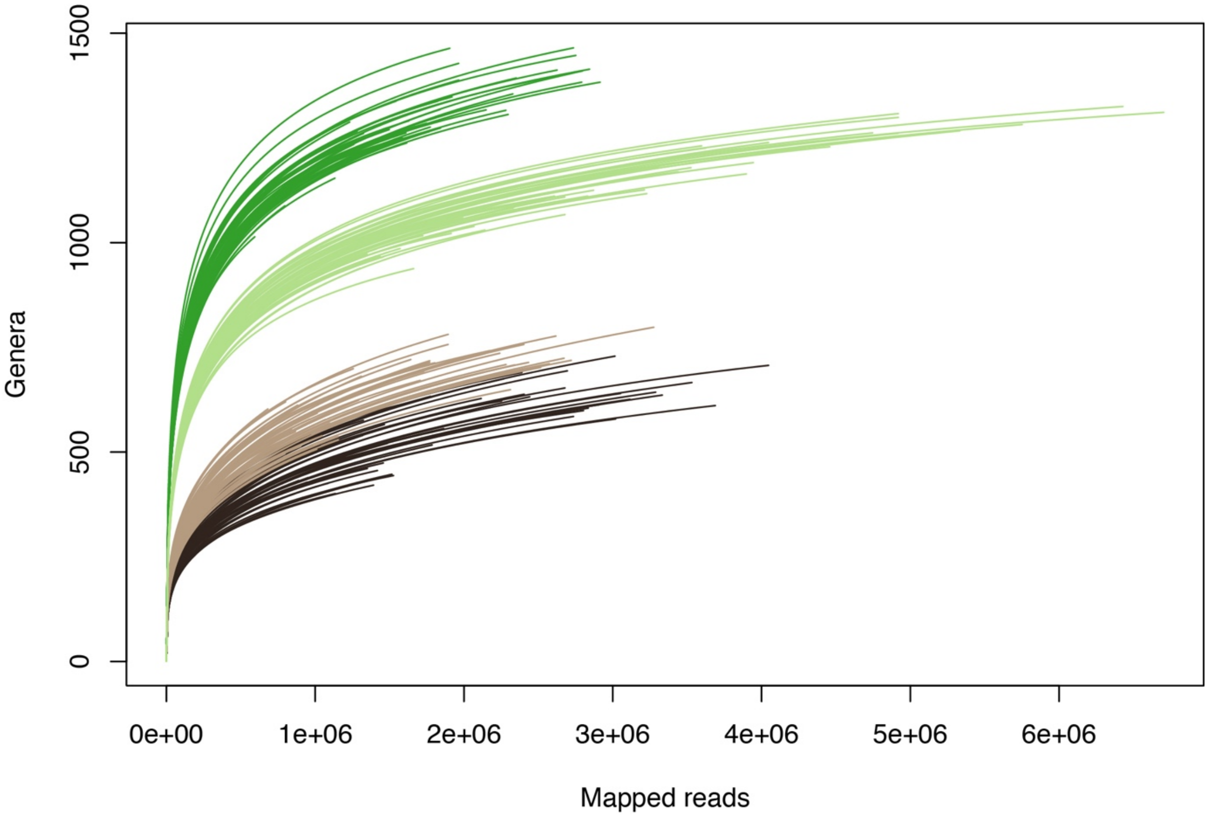

Figure S2: Rarefaction curves for all samples from pig feces P1 (dark brown), pig feces P2 (light brown), sewage S1 (dark green), and sewage S2 (light green).

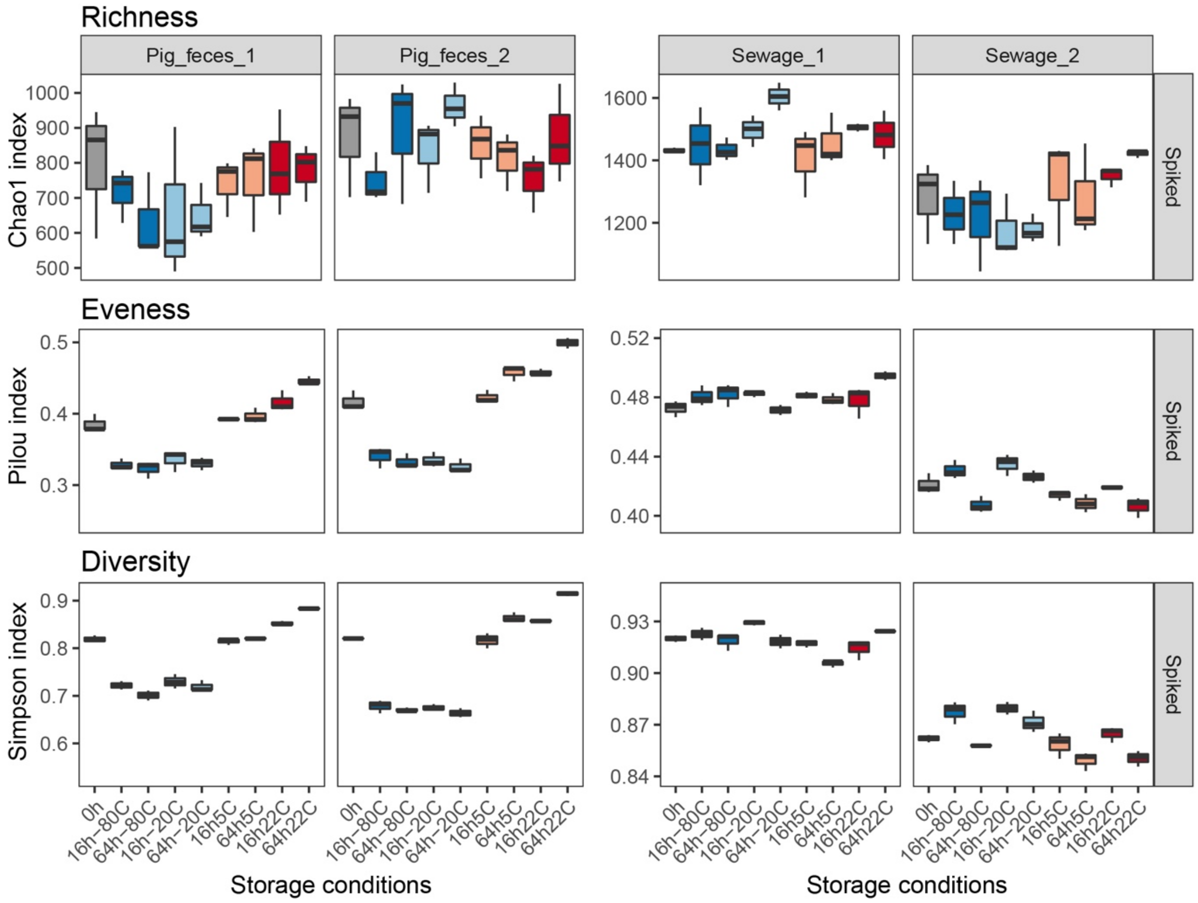

Figure S3: Alpha diversity for samples spiked with a mock community: richness (Chao1), evenness (Pielou's evenness), and diversity (Simpson). The indices were calculated from the count table aggregated at genus level. For the results of the individual unspiked samples, see Figure S1.

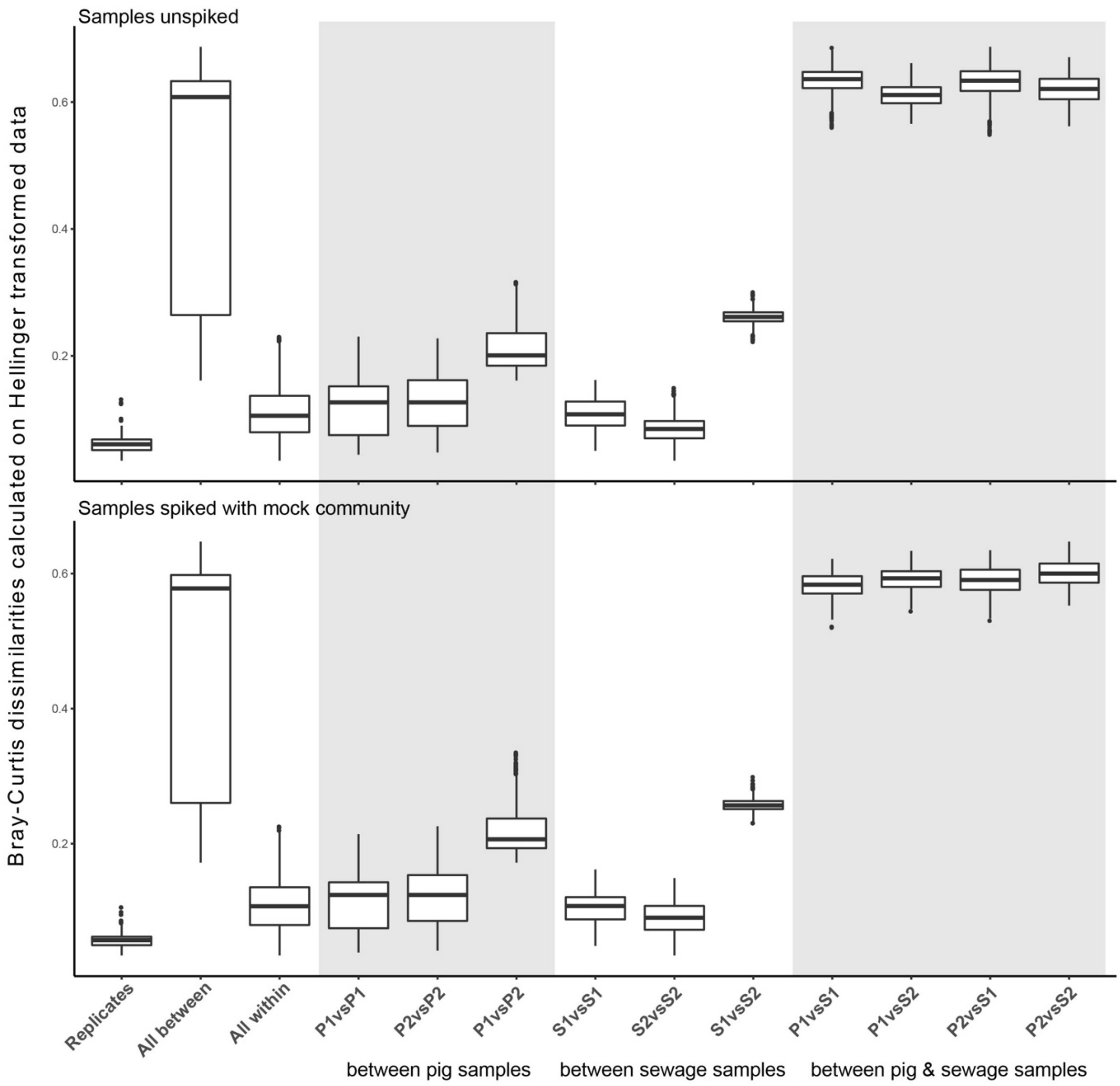

Figure S4: Boxplots of Bray Curtis dissimilarities calculated on Hellinger transformed data. Dissimilarities were grouped according to within sample comparisons and between all possible sample comparison combinations, for unspiked and spiked samples, respectively. Additional boxplots were made for all within and between sample comparisons as well as comparisons between replicates.

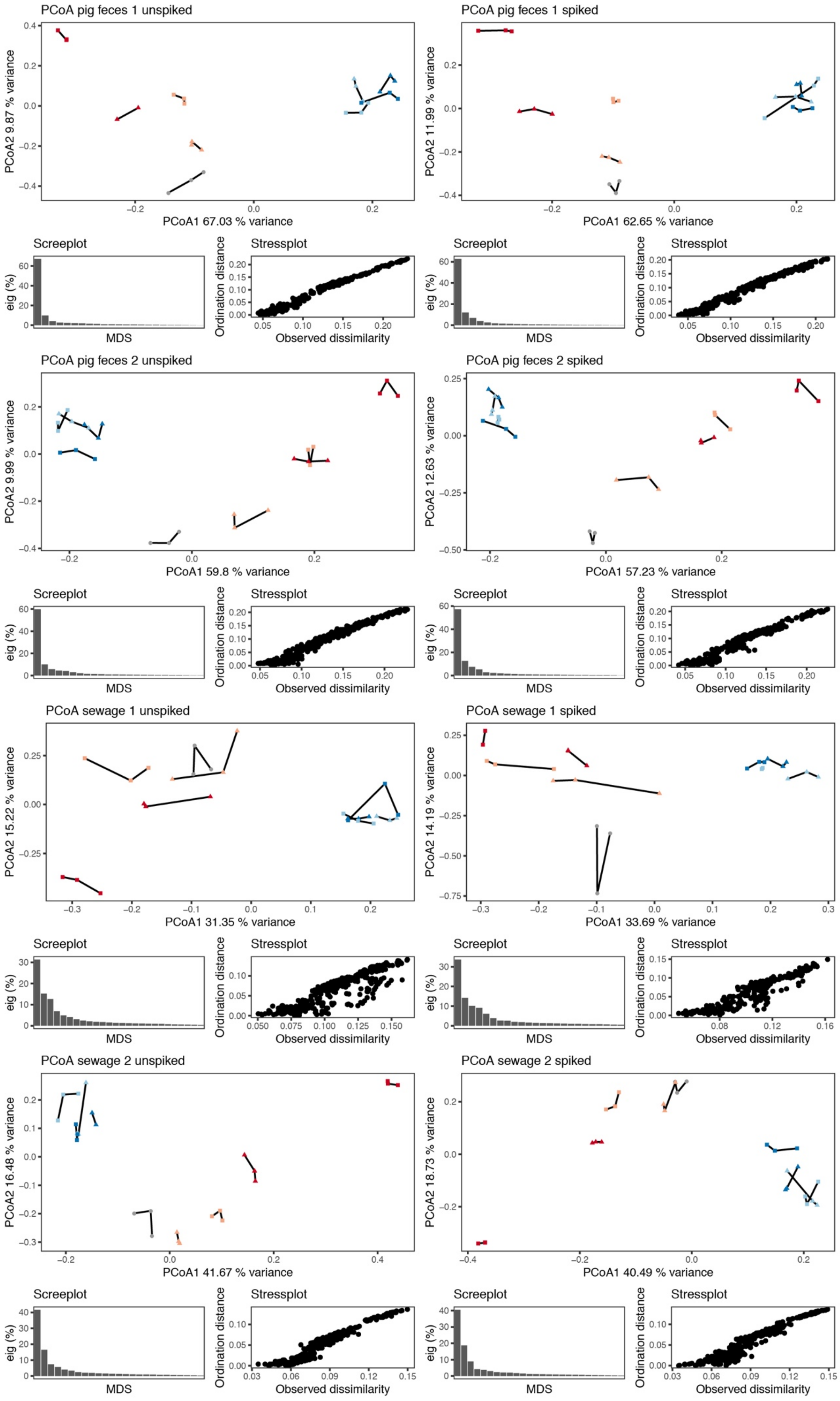
PCoA pig feces 1 unspiked
PCoA2 9.87 % variance
PCoA1 67.03 % variance
PCoA pig feces 1 spiked
PCoA2 11.99 % variance
PCoA1 62.65 % variance
Screeplot
eig (%)
MDS
Stressplot
Ordination distance
Observed dissimilarity
PCoA pig feces 2 unspiked
PCoA2 9.99 % variance
PCoA1 59.8 % variance
PCoA pig feces 2 spiked
PCoA2 12.63 % variance
PCoA1 57.23 % variance
PCoA sewage 1 unspiked
PCoA2 15.22 % variance
PCoA1 31.35 % variance
PCoA sewage 1 spiked
PCoA2 14.19 % variance
PCoA1 33.69 % variance
PCoA sewage 2 unspiked
PCoA2 16.48 % variance
PCoA1 41.67 % variance
PCoA sewage 2 spiked
PCoA2 18.73 % variance
PCoA1 40.49 % variance

Figure S5: Principal coordinates analysis (PCoA) for each individual sample both unspiked and spiked. Bray-Curtis dissimilarities were calculated on Hellinger transformed data. The vegan function capscale was used to perform PCoA on the dissimilarity matrix. Variance explained by the first two dimensions are included at the axes, respectively. Validation plots (screeplots and stressplots) are included below for each of the corresponding PCoA plot, respectively.

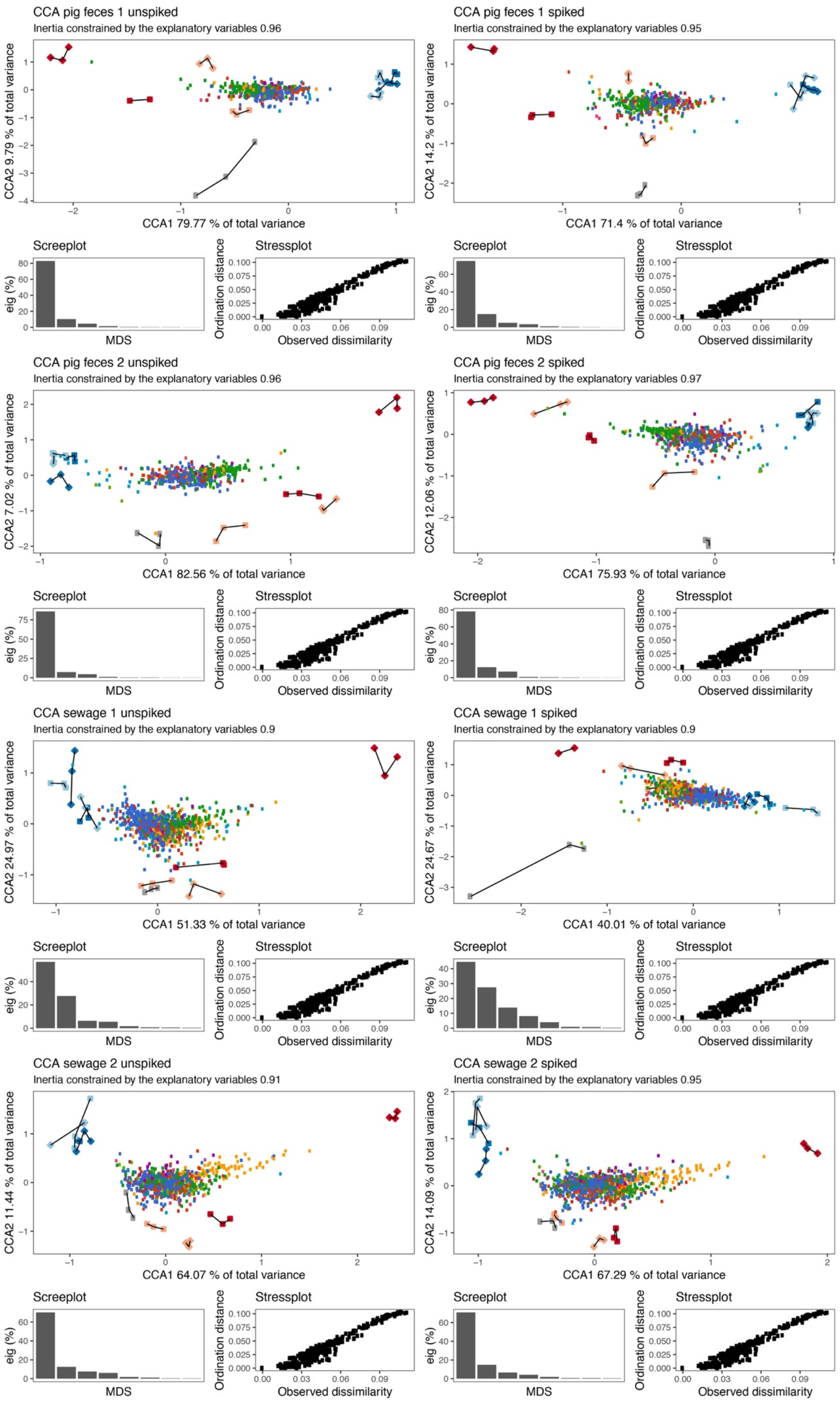
CCA pig feces 1 unspiked
Inertia constrained by the explanatory variables 0.96
CCA2 9.79 % of total variance
CCA1 79.77 % of total variance
CCA pig feces 1 spiked
Inertia constrained by the explanatory variables 0.95
CCA2 14.2 % of total variance
CCA1 71.4 % of total variance
Screeplot
eig (%)
MDS
Stressplot
Ordination distance
Observed dissimilarity
Screeplot
eig (%)
MDS
Stressplot
Ordination distance
Observed dissimilarity
CCA pig feces 2 unspiked
Inertia constrained by the explanatory variables 0.96
CCA2 7.02 % of total variance
CCA1 82.56 % of total variance
CCA pig feces 2 spiked
Inertia constrained by the explanatory variables 0.97
CCA2 12.06 % of total variance
CCA1 75.93 % of total variance
Screeplot
eig (%)
MDS
Stressplot
Ordination distance
Observed dissimilarity
Screeplot
eig (%)
MDS
Stressplot
Ordination distance
Observed dissimilarity
CCA sewage 1 unspiked
Inertia constrained by the explanatory variables 0.9
CCA2 24.97 % of total variance
CCA1 51.33 % of total variance
CCA sewage 1 spiked
Inertia constrained by the explanatory variables 0.9
CCA2 24.67 % of total variance
CCA1 40.01 % of total variance
Screeplot
eig (%)
MDS
Stressplot
Ordination distance
Observed dissimilarity
Screeplot
eig (%)
MDS
Stressplot
Ordination distance
Observed dissimilarity
CCA sewage 2 unspiked
Inertia constrained by the explanatory variables 0.91
CCA2 11.44 % of total variance
CCA1 64.07 % of total variance
CCA sewage 2 spiked
Inertia constrained by the explanatory variables 0.95
CCA2 14.09 % of total variance
CCA1 67.29 % of total variance
Screeplot
eig (%)
MDS
Stressplot
Ordination distance
Observed dissimilarity
Screeplot
eig (%)
MDS
Stressplot
Ordination distance
Observed dissimilarity

Figure S6: Canonical correspondence analysis (CCA) of each individual samples both unspiked and spiked exploring the taxonomic microbial community composition (coloured points) constrained by the storage conditions (coloured shapes; replicates are connected with lines). The vegan function cca was used to perform CCA on the count matrix. The variance explained by the first two dimensions is included at the axes, respectively. Validation plots (screeplots and stressplots) are included below for each of their corresponding CCA plot, respectively.

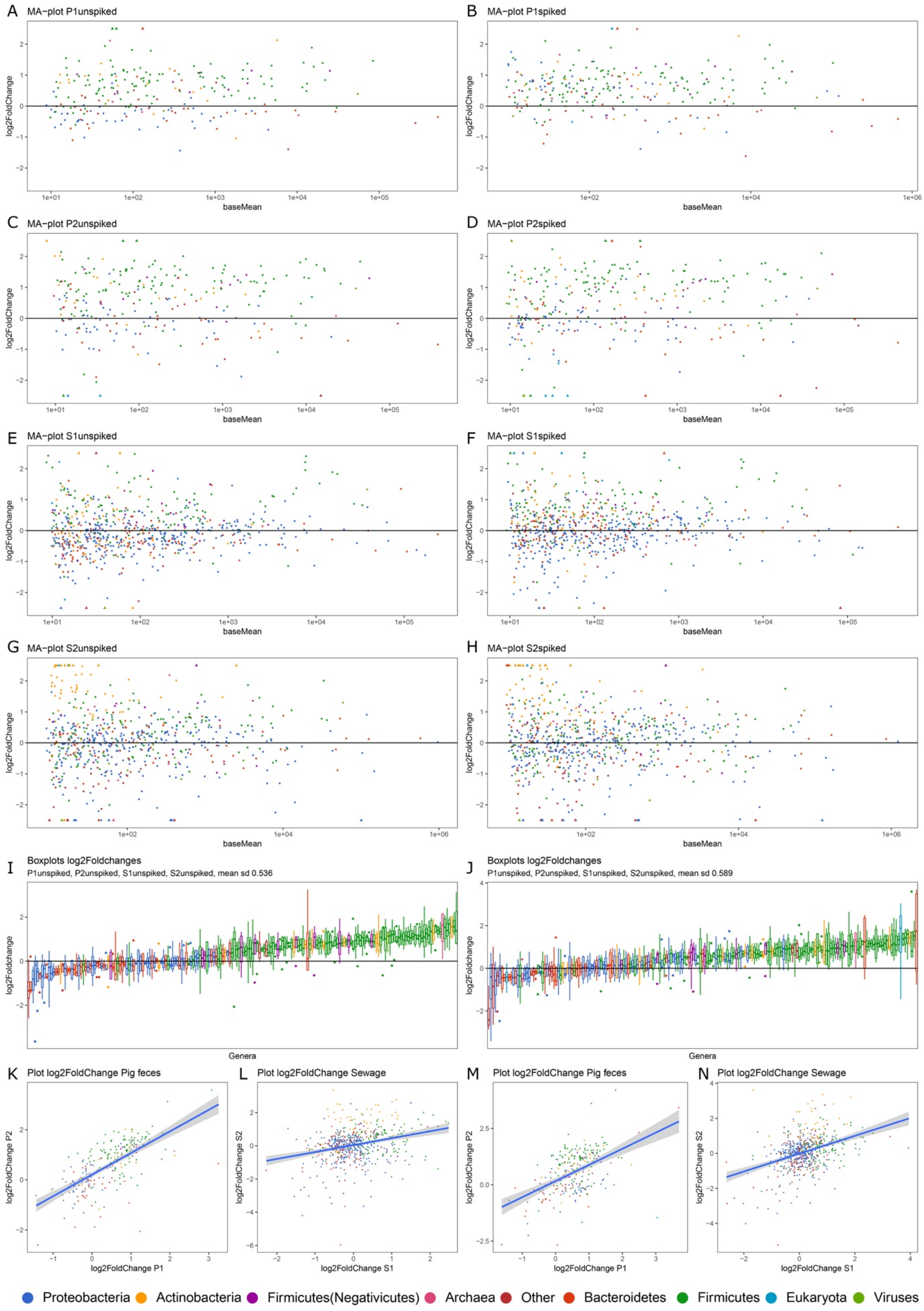
A
MA−plot P1unspiked
B
MA−plot P1spiked
C
MA−plot P2unspiked
D
MA−plot P2spiked
E
MA−plot S1unspiked
F
MA−plot S1spiked
G
MA−plot S2unspiked
H
MA−plot S2spiked
log2FoldChange
baseMean
I
Boxplots log2Foldchanges
P1unspiked, P2unspiked, S1unspiked, S2unspiked, mean sd 0.536
J
Boxplots log2Foldchanges
P1unspiked, P2unspiked, S1unspiked, S2unspiked, mean sd 0.589
log2Foldchange
Genera
K
Plot log2FoldChange Pig feces
L
Plot log2FoldChange Sewage
M
Plot log2FoldChange Pig feces
N
Plot log2FoldChange Sewage
log2FoldChange P2
log2FoldChange P1
log2FoldChange S2
log2FoldChange S1
Proteobacteria
Actinobacteria
Firmicutes(Negativicutes)
Archaea
Other
Bacteroidetes
Firmicutes
Eukaryota
Viruses

Figure S7: Taxonomic microbial community patterns comparing two storage conditions - immediate DNA extraction & storage at 22°C for 64h. The MA-plots are indicating differentially abundant genera (A-H). Boxplots of log2-fold changes of all shared organisms in all samples sorted from lowest to highest mean (I and J). Scatterplots of log2-fold changes of all shared genera between the two pig fecal samples and two sewage samples (K-N). Plots are generated for both unspiked and spiked sets of samples. Positive log2-fold changes specify a higher relative abundance in samples stored at 22°C for 64h relative to 0 h (A-N). The colour code for the taxonomic groups is indicated at the bottom.

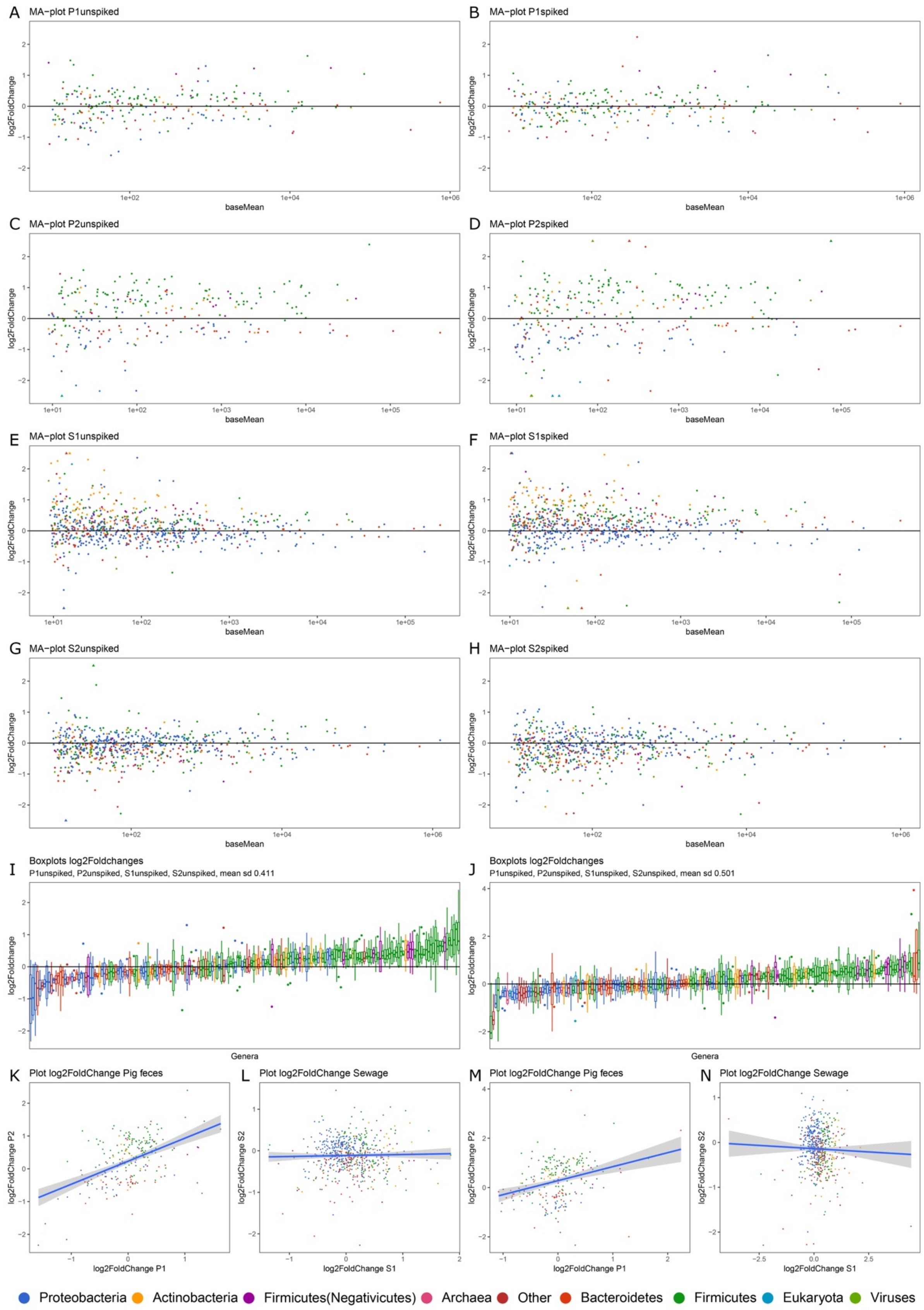

Figure S8: Taxonomic microbial community patterns comparing two storage conditions - immediate DNA extraction & storage at 5°C for 64h. The MA-plots are indicating differentially abundant genera (A-H). Boxplots of log2-fold changes of all shared organisms in all samples sorted from lowest to

highest mean (I and J). Scatterplots of log2-fold changes of all shared genera between the two pig fecal samples and two sewage samples (K-N). Plots are generated for both unspiked and spiked sets of samples. Positive log2-fold changes specify a higher relative abundance in samples stored at 5°C for 64h relative to 0 h (A-N). The colour code for the taxonomic groups is indicated at the bottom.

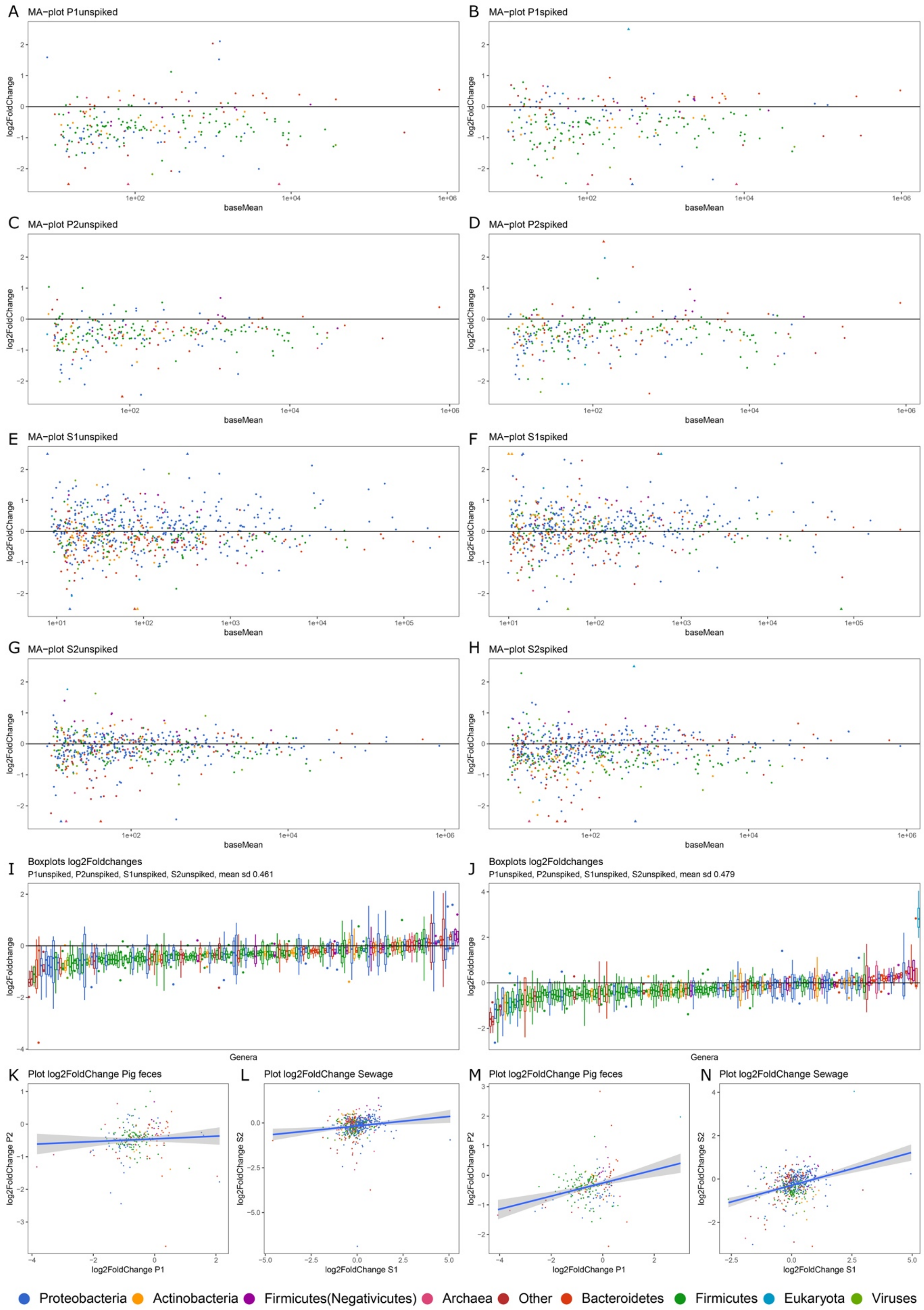

Figure S9: Taxonomic microbial community patterns comparing two storage conditions - immediate DNA extraction & storage at -80°C for 64h. The MA-plots are indicating differentially abundant genera (A-H). Boxplots of log2-fold changes of all shared organisms in all samples sorted from lowest to

highest mean (I and J). Scatterplots of log2-fold changes of all shared genera between the two pig fecal samples and two sewage samples (K-N). Plots are generated for both unspiked and spiked sets of samples. Positive log2-fold changes specify a higher relative abundance in samples stored at -80°C for 64h relative to 0 h (A-N). The colour code for the taxonomic groups is indicated at the bottom.

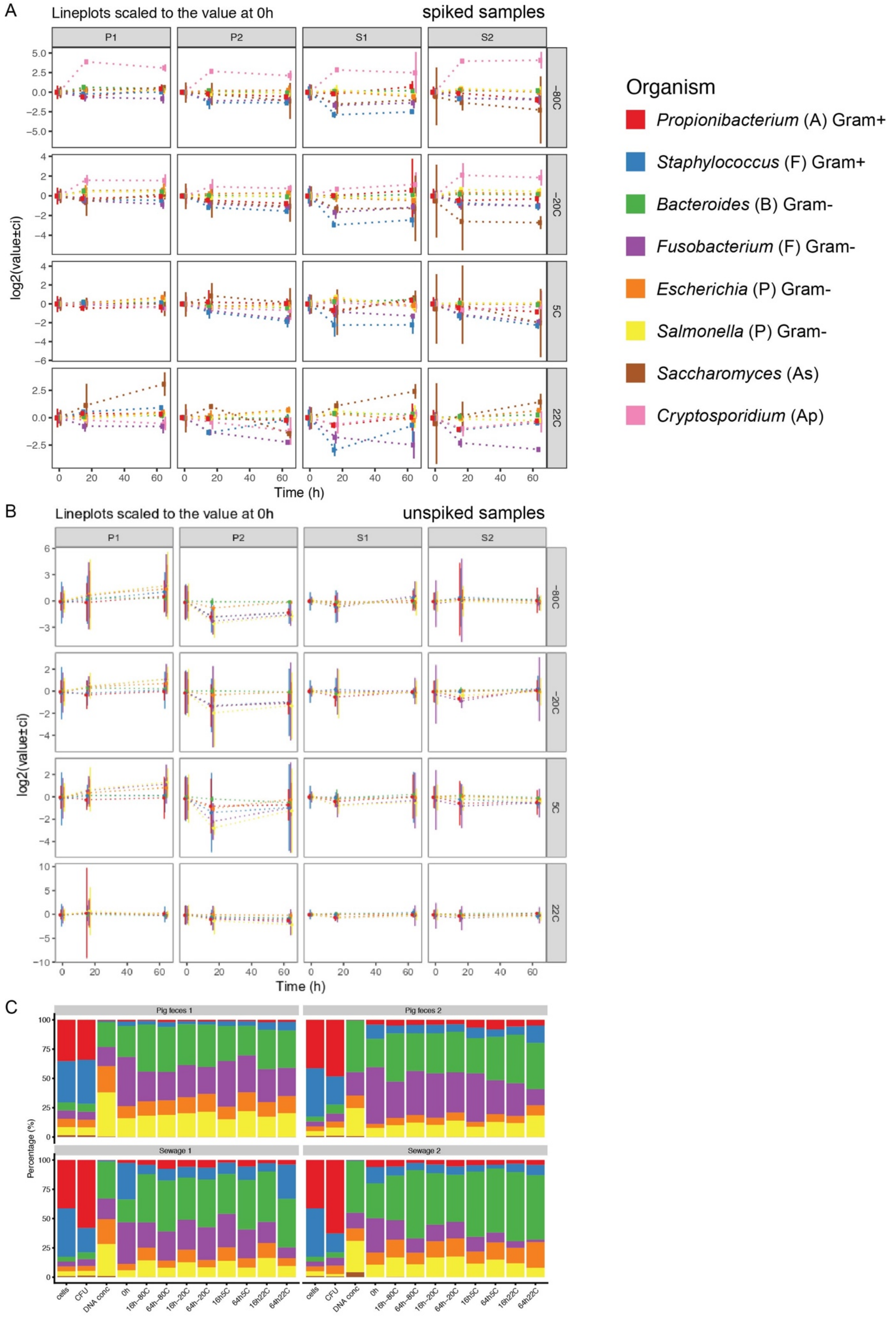
A
Lineplots scaled to the value at 0h
spiked samples
P1
P2
S1
S2
-80C
-20C
5C
22C
log2(value±ci)
Time (h)
B
Lineplots scaled to the value at 0h
unspiked samples
P1
P2
S1
S2
-80C
-20C
5C
22C
log2(value±ci)
Time (h)
C
Pig feces 1
Pig feces 2
Sewage 1
Sewage 2
Percentage (%)
cells
CFU
DNA conc
0h
16h-80C
64h-80C
16h-20C
64h-20C
16h5C
64h5C
16h22C
64h22C
Organism
Propionibacterium (A) Gram+
Staphylococcus (F) Gram+
Bacteroides (B) Gram-
Fusobacterium (F) Gram-
Escherichia (P) Gram-
Salmonella (P) Gram-
Saccharomyces (As)
Cryptosporidium (Ap)

Figure S10: Abundance of the mock community members. (A+B) Lineplots of the mock community organisms in spiked (A) and unspiked (B) samples. The mock community members are represented at genus level namely *Propionibacterium, Staphylococcus, Bacteroides, Fusobacterium, Escherichia, Salmonella, Saccharomyces*, and *Cryptosporidium*. The values were scaled to the average abundance obtained at timepoint 0h. (C) In the first three columns, bacterial cell counts, colony forming units (CFU) and DNA concentration represent the compositions estimated from all cells counted by microscopy in a Petroff counting chamber, viable cells via counting colony-forming units through cultivation, and DNA concentration of the DNA extracts obtained from the individual organisms using the DNA isolation protocol for microbiome samples. The remaining stacked bars represent the microbial community composition at the different storage conditions based on read counts. The read counts represent averages normalized according to genome size and from which the background levels of reads were removed using the information from the unspiked samples.

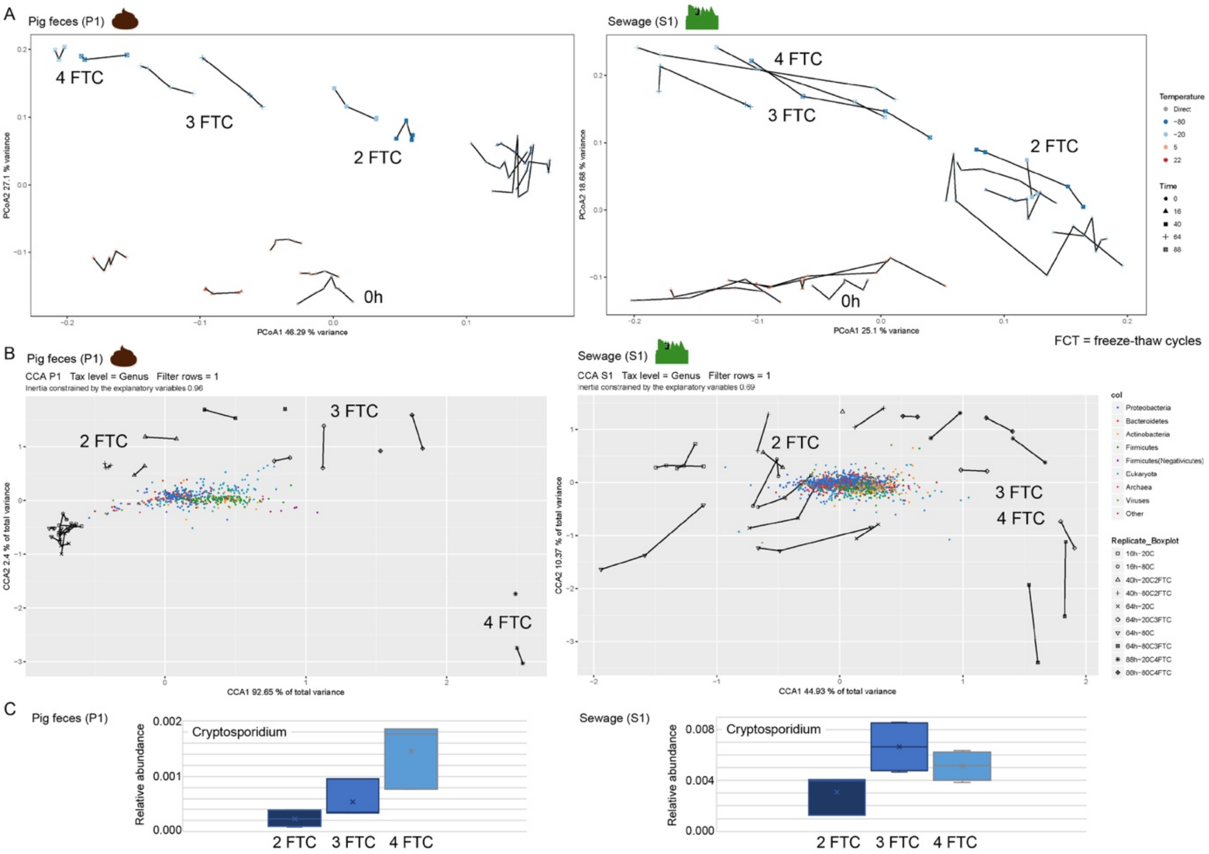

Figure S11: Microbiome patterns upon repeated freeze-thaw cycles. (A) Principal coordinates analysis (PCoA) for pig feces P1 and sewage S1. Bray-Curtis dissimilarities were calculated on Hellinger transformed data. The vegan function capscale was used to perform PCoA on the dissimilarity matrix. Variance explained by the first two dimensions are included at the axes, respectively. (B) Canonical correspondence analysis (CCA) of pig feces P1 and sewage S1 exploring the taxonomic microbial community composition (coloured points) constrained by the storage conditions (coloured shapes; replicates are connected with lines). The vegan function cca was used to perform CCA on the count matrix. The variance explained by the first two dimensions is included at the axes, respectively. (C) The relative abundance of Cryptosporidium upon series of freeze-thaw cycles. The number of freeze-thaw cycles is indicates in the plots.

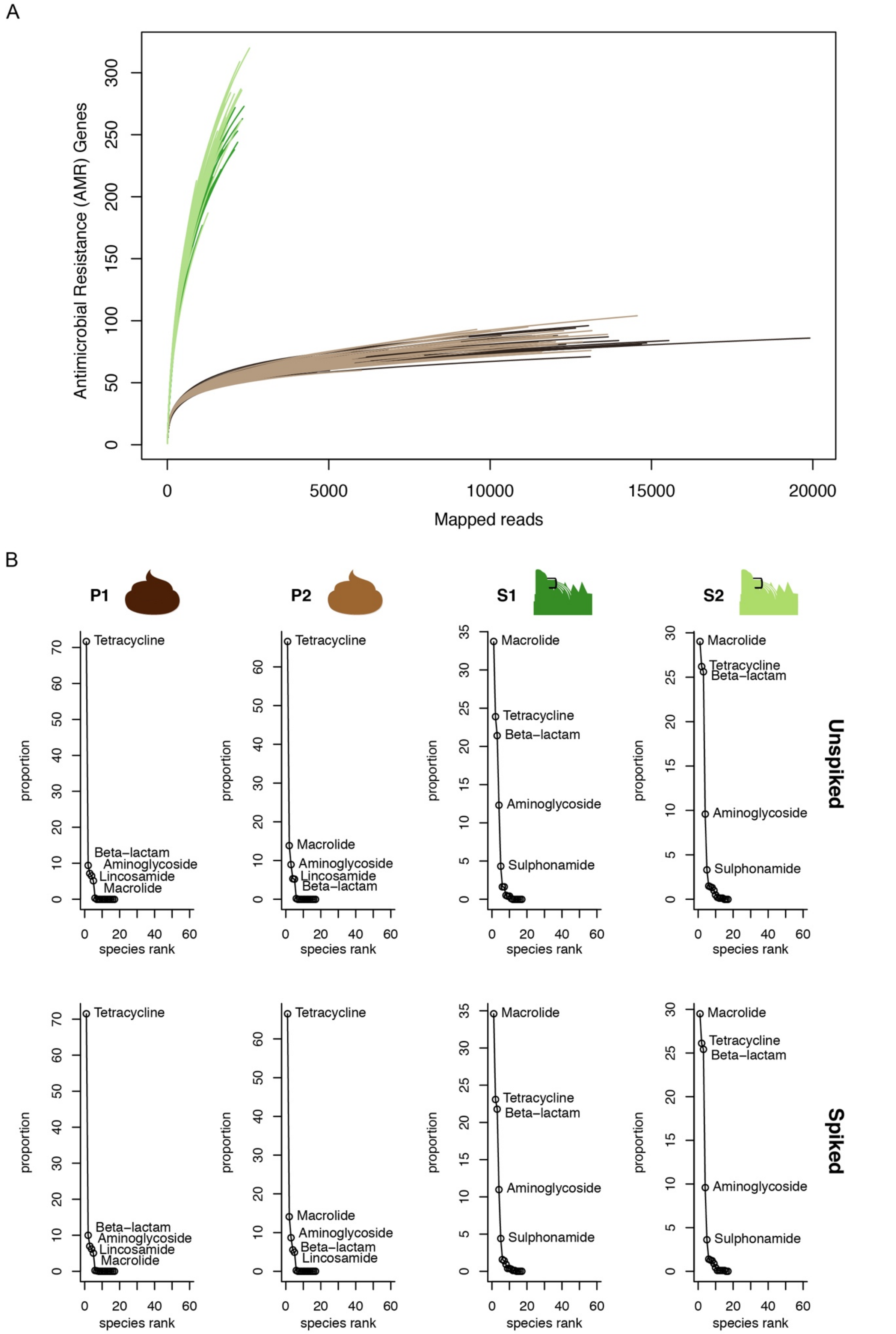
A
Antimicrobial Resistance (AMR) Genes
Mapped reads
B
P1
P2
S1
S2
Unspiked
Spiked
proportion
species rank
Tetracycline
Beta–lactam
Aminoglycoside
Lincosamide
Macrolide
Sulphonamide

Figure S12: Antimicrobial resistance (AMR) abundance. (A) Rarefaction curves for AMR genes. (B) Rank abundance curves of resistance gene classes showing their abundance distribution in the different samples for both unspiked and spiked pig feces (P1, P2) and sewage (S1, S2).

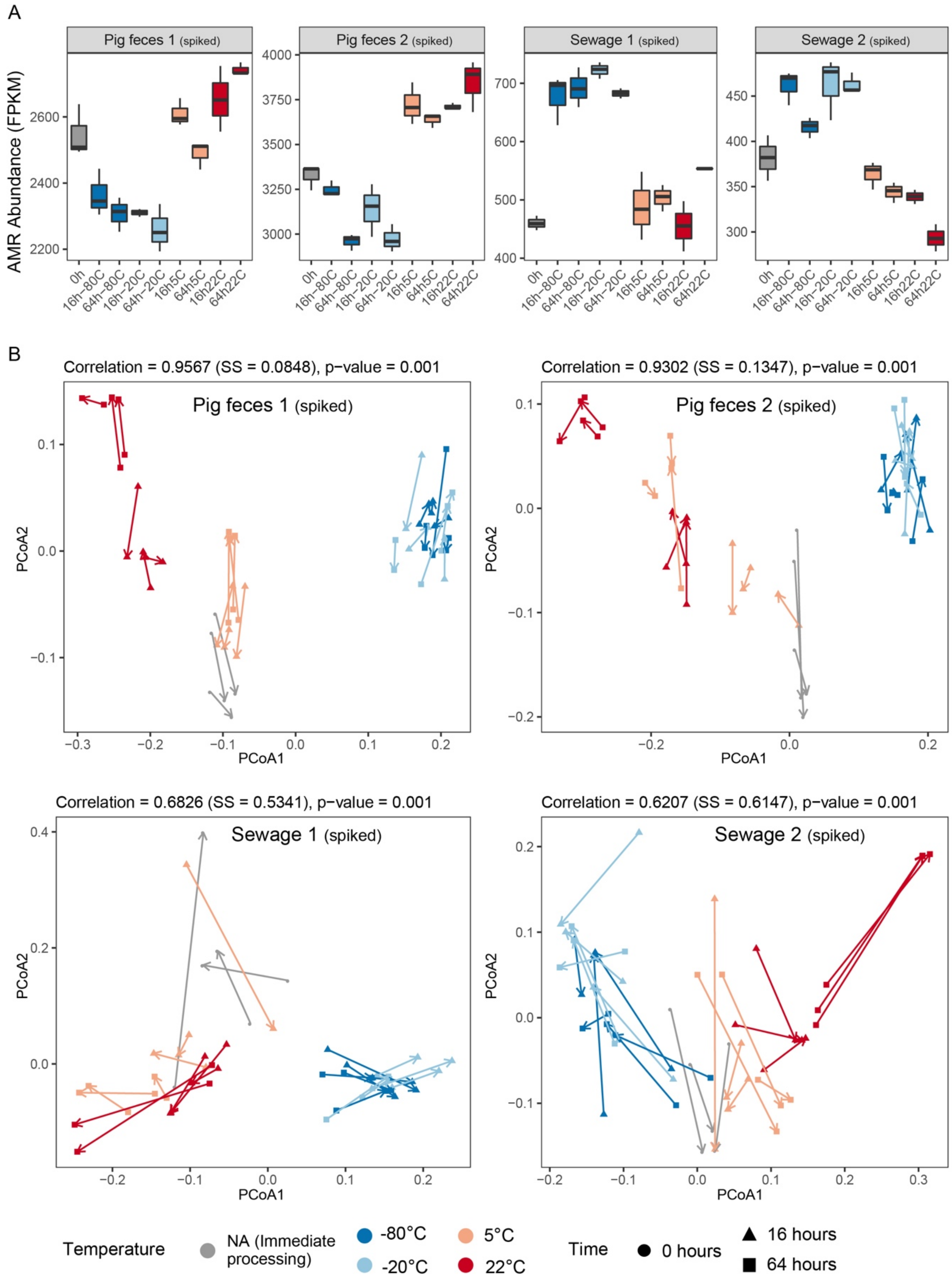

Figure S13: The effect of sample storage conditions on the resistome. A) Boxplots displaying total antimicrobial resistance (AMR) abundance in the spiked samples (P1, P2, S1, S2) measured in FPKM relative to the total number of bacterial reads. B) Procrustes rotation comparing the resistome and taxonomic dissimilarities in the four spiked samples. For the results of the unspiked samples, see Figure 5 in the main text.

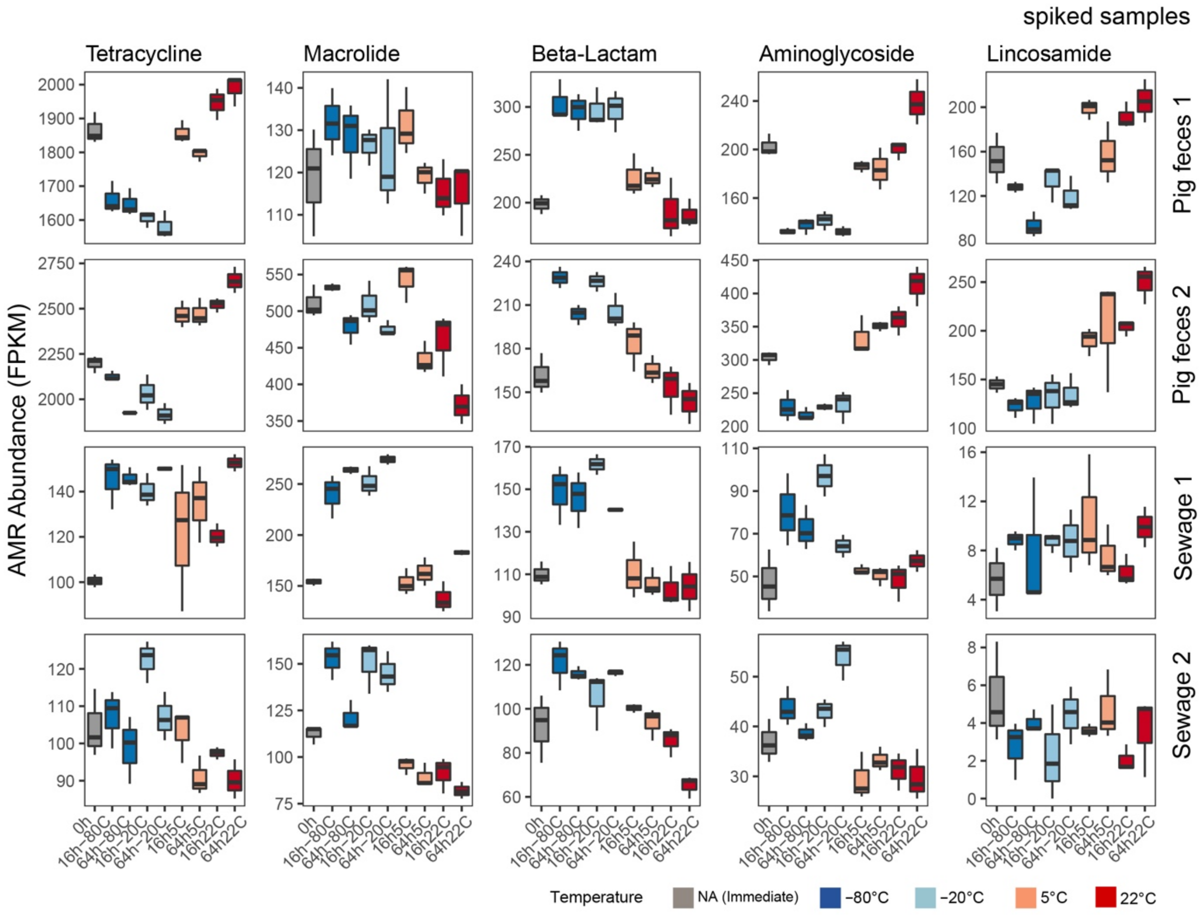

Figure S14: The effect of storage on the abundance of antimicrobial resistance classes. Boxplots displaying total antimicrobial resistance (AMR) abundance for the most abundant antimicrobial resistance classes in the spiked samples (P1, P2, S1, S2). The abundance was measured in FPKM relative to the total number of bacterial reads. For the results of the unspiked samples, see Figure 6 in the main text.
